# Extreme Recombination Frequencies Shape Genome Variation and Evolution in the Honeybee, *Apis mellifera*

**DOI:** 10.1371/journal.pgen.1005189

**Published:** 2015-04-22

**Authors:** Andreas Wallberg, Sylvain Glémin, Matthew T. Webster

**Affiliations:** 1 Department of Medical Biochemistry and Microbiology, Science for Life Laboratory, Uppsala University, Uppsala, Sweden; 2 Institut des Sciences de l’Evolution (ISEM—UMR 5554 Université de Montpellier-CNRS-IRD-EPHE), France; 3 Department of Ecology and Genetics, Evolutionary Biology Centre, Uppsala University, Uppsala, Sweden; Institute of Science and Technology Austria (IST Austria), AUSTRIA

## Abstract

Meiotic recombination is a fundamental cellular process, with important consequences for evolution and genome integrity. However, we know little about how recombination rates vary across the genomes of most species and the molecular and evolutionary determinants of this variation. The honeybee, *Apis mellifera*, has extremely high rates of meiotic recombination, although the evolutionary causes and consequences of this are unclear. Here we use patterns of linkage disequilibrium in whole genome resequencing data from 30 diploid honeybees to construct a fine-scale map of rates of crossing over in the genome. We find that, in contrast to vertebrate genomes, the recombination landscape is not strongly punctate. Crossover rates strongly correlate with levels of genetic variation, but not divergence, which indicates a pervasive impact of selection on the genome. Germ-line methylated genes have reduced crossover rate, which could indicate a role of methylation in suppressing recombination. Controlling for the effects of methylation, we do not infer a strong association between gene expression patterns and recombination. The site frequency spectrum is strongly skewed from neutral expectations in honeybees: rare variants are dominated by AT-biased mutations, whereas GC-biased mutations are found at higher frequencies, indicative of a major influence of GC-biased gene conversion (gBGC), which we infer to generate an allele fixation bias 5 – 50 times the genomic average estimated in humans. We uncover further evidence that this repair bias specifically affects transitions and favours fixation of CpG sites. Recombination, via gBGC, therefore appears to have profound consequences on genome evolution in honeybees and interferes with the process of natural selection. These findings have important implications for our understanding of the forces driving molecular evolution.

## Author Summary

Evolution results from changes in allele frequencies in populations. The main forces that cause such changes are natural selection and random genetic drift. However, an additional process, GC-biased gene conversion (gBGC), associated with meiotic recombination, affects the probability that alleles are passed from one generation to the next. The honeybee, *Apis mellifera*, has extremely high recombination rates—more than 20 times to those observed in humans. However, the reason for this is unknown and the effects of such high recombination rates on evolution are not well understood. Here we use patterns of genetic variation in the genomes of 30 honeybees to infer variation in the rate of recombination across the genome. We find that recombination rates and levels of genetic variation are strongly correlated, which is indicative of a pervasive impact of natural selection on genetic variation. We also infer a major role of DNA methylation in determining recombination rates in genes. Patterns of genetic variation appear to be strongly skewed due to the effects of gBGC, suggesting that recombination generates a bias in transmission of alleles during meiosis. This process seems to be interfering with the efficacy of selection at removing deleterious alleles and favouring beneficial ones. Recombination therefore has a huge impact on genetic variation and evolution in honeybees and appears to play a dominant role in genome evolution.

## Introduction

In most sexual eukaryotes, average recombination rates do not greatly exceed one crossover per chromosome arm, which is commonly a minimum requirement for correct meiosis [1]. However, the honeybee, *Apis mellifera*, has extremely high recombination rates, averaging 19–37 cM/Mb [2–4], which corresponds to more than 5 crossovers per chromosome pair per meiosis. Such high rates are observed in other social insects but not their solitary cousins [5,6]. This suggests that high recombination rates are an adaptation favoured by eusociality although the specific causes are unknown. Insight into this question can be gained by analysing the fine-scale landscape of recombination rate variation in order to understand the molecular mechanisms that govern it.

The molecular mechanisms that determine the genomic distribution of recombination events in honeybees are unclear. In a wide range of species, recombination events are strongly clustered into short hotspots a few kb in length [7–10]. In human and mouse, these hotspots are found to be enriched for a DNA motif recognised by the protein PRDM9 [11–14]. This protein binds to the DNA motif and catalyses a histone modification that acts as a mark for the formation of a DNA double stranded break in the same location [15]. In species without an active PRDM9, hotspots are often present, but other features may define them. For example, in dog, where PRDM9 is inactive, recombination events are clustered in un-methylated CpG islands [16–18]. In yeast and *Arabidopsis* recombination hotspots are observed in nucleosome-depleted open chromatin and gene promoters [8,9].

The few invertebrate genomes analysed so far tend to lack extreme recombination hotspots [19,20]. In particular, recombination rates in the fruit fly *Drosophila melanogaster* appear to be less variable across the genome than other species where fine-scale genetic maps are available [20–23]. Genetic maps of the honeybee do not indicate the presence of hotspots with extremely elevated rates [2,3,24] or the presence of enriched sequence motifs [4]. This is consistent with the absence of a PRDM9-like mechanism controlling recombination rates in insects and suggests that other factors are more important. One such factor could be DNA methylation. Unlike fruit flies, the honeybee has an intact methylation system [25,26]. It is therefore possible that rates of recombination in the honeybee genome are influenced by DNA methylation patterns, as observed in some other taxa [16–18,27].

In a diverse range of species, local rates of crossing-over correlate with genetic diversity but not with genetic divergence [28,29]. These correlations are inferred to be due to an indirect effect of recombination due to the interaction between selection and linkage and their strength can be used to make inferences about the pervasiveness of natural selection. Positive selection on favourable mutations or negative selection against deleterious changes reduce levels of linked variation by the processes of genetic hitchhiking and background selection and these effects are predicted to be larger in regions of low recombination [30,31], resulting in lower genetic diversity in these regions. Strong correlations exist in many species of fruit fly that have been used to predict that large proportions of the genome are affected by selection [32–34], whereas in humans such correlations are weaker [35,36], suggesting a less pervasive impact of selection on genetic variation. Social insects such as honeybees have lower effective population sizes than solitary ones [37,38] and it is unclear if selection has a similarly pervasive impact on genome variation.

A number of hypotheses have been proposed to explain the extremely high recombination rates in honeybees and other social insects. One class of hypotheses suggests that they represent an adaptation important for the evolution of behavioural phenotypes in the worker caste. This could be because the evolution of eusociality entailed rapid evolution and specialisation of workers [39]. Alternatively, high intra-colony variability in worker phenotypes could be beneficial because it results in a more efficient workforce [40,41]. These factors could lead to increased recombination rates in the vicinity of genes specifically involved in worker phenotypes. Some studies have reported evidence for higher recombination rates in genes with worker-biased expression [4,39]. However, the cause of these associations is unclear and several questions remain. In particular, it is not known whether worker-biased genes are preferentially located in regions of high recombination, or whether there is a direct influence of gene expression or a related process on recombination rate within genes.

Recombination can have profound affects of genome evolution via GC-biased gene conversion (gBGC; reviewed in [42]). This process is believed to occur due to the biased repair of nucleotide mismatches that occur in heteroduplex DNA generated from pairing of two alleles during meiotic recombination. This involves a small bias towards repairing a mismatch involving a G/C (or S, for strong) nucleotide paired with an A/T (or W, for weak) nucleotide in favour of retaining the S allele, which results in an increased probability of transmitting the S allele into the gametes. There is a large amount of indirect evidence that this process occurs, indicating that genomic regions of high recombination accumulate GC-biased nucleotide substitutions over evolutionary time [43,44], which results in a correlation between recombination and GC content [45]. A transmission bias towards S alleles has also been directly observed in yeast [46] by analysis of the products of meiosis and humans [47] by analysing transmission through pedigrees.

The population dynamics of gBGC are equivalent to selection acting to increase the fixation probability of weak-to-strong (WS) mutations [48]. As such, gBGC can have effects on the site frequency spectrum [49–51] and rate of nucleotide substitution [52–54] similar to selection. It can also interfere with the process of natural selection. For example, gBGC could cause increased substitution rates in functional regions that can be mistaken for positive selection [52–54]. It can also lead to fixation of deleterious changes, including those underlying genetic disease in humans [55,56]. The transmission bias caused by gBGC also results in a skewed allele frequency spectrum, where WS mutations segregate at higher frequencies. Glémin et al. [57] modelled this property to estimate the strength of gBGC in the human population. The average strength of gBGC, *B*, was estimated as 0.38, (where *B* = 4*N*_*E*_ *b*, *N*_*E*_ being the effective population size and *b* the gBGC coefficient) but 1% to 2% of the genome was estimated to be subject to strong gBGC with *B*>5. Phylogenetic estimates indicate variation in *B* over two orders of magnitude among placental mammals [58]. The extreme recombination rates of honeybees could also indicate that gBGC is also very powerful, suggesting it could significantly impact molecular evolution in honeybees. In support of this previous studies found elevated frequencies of WS mutations, particularly in regions of high GC content [2,39].

A striking and unique feature of the honeybee genome is the over-representation of CpG dinucleotides [26]. The statistic CpG_O/E_ measures the frequency of CpG dinucleotides in a nucleotide sequence compared to its expected value based on individual frequencies of Cs and Gs. In species where most CpG sites in the genome are methylated, as is the case in plants and vertebrates, CpG sites occur at a much lower frequency than expected due to the effects of methylated CpG hypermutability (average CpG_O/E_ in humans is 0.2) and this value rarely exceeds one in eukaryote genomes. The honeybee genome is unique in that it has a much higher frequency of CpG sites than expected (CpG_O/E_ is around 1.67). The reason for this is unclear, but possible explanations are a mutational bias in favour of CpG sites or a fixation bias due to gBGC that favours the fixation of mutations that generate CpG dinucleotides.

There are a number of unresolved questions regarding the evolution, molecular control and consequences of recombination in the honeybee genome. Firstly, is there evidence for recombination hotspots? How does gene expression and DNA methylation affect local rates of recombination? The answer to these questions could give us insight into how recombination is controlled in invertebrates. Secondly, does recombination modulate strength of natural selection across the genome? This can be addressed by investigating the correlation between recombination rate and the levels of genetic diversity and divergence. Thirdly, is there evidence for a local increase in recombination rate in the vicinity of genes with worker-biased expression? It has been suggested that this could be selectively advantageous due to the importance of worker phenotypes in the evolution of eusociality. Finally, what effects do the extremely high levels of recombination in honeybee have on the strength of gBGC? How does gBGC impact genome variation and the frequency of CpG sites in the genome? We can address these questions by analysing the shape of the site frequency spectrum for different SNP categories and estimating the value of *B*.

Here we construct a fine-scale map of recombination rate variation honeybee using population-scale resequencing dataset [37] with the aim of addressing these questions. Our estimates show good correspondence with a previous genetic map [3]. Compared to the human genome, recombination events do not appear to be strongly partitioned into hotspots in the honeybee genome. Our data is consistent with an effect of germline methylation generating variation in crossover rate by suppressing recombination. We find evidence for a strong association between recombination and levels of genetic variation. In contrast to previous studies, we do not find that worker-biased expression is a strong predictor of high recombination rate compared to other factors. We also uncover a major effect of recombination on genome variation via the process of gBGC, which is stronger than observed in any other species and has a major impact on genome variation and evolution.

## Results

### Construction of an LD-based map of recombination rate variation

We constructed a high-resolution map of rates of crossing-over from patterns of linkage disequilibrium among 6.2 million SNPs observed across 60 copies of the sixteen nuclear honeybee chromosomes. We chose to use samples from Africa, sequenced as part of a larger study, because they are the most genetically diverse and because there is no evidence for population structure between them [37]. We used the LDhat method, which estimates the population-scaled recombination rate *ρ* across the genome. This is related to the recombination frequency *r* by the equation *ρ* = 3*N*_*E*_ *r* in the case of haplodiploid species, where *N*_*E*_ is effective population size. The LD map contained 306,764 discrete rate intervals. About 50% of the genome is covered by intervals of 5 kb or longer in this map. The mean recombination rate is 390 *ρ*/kb and the average rate change is ~9% between adjacent intervals. Scaling by a *N*_*E*_ of 500,000, estimated previously using the same set of African samples [37], this corresponds to an average crossover frequency, *r*, of 26.0 cM/Mb, which is in agreement with previous estimates of 19–37 cM/Mb [2–4].

**Fig 1:**
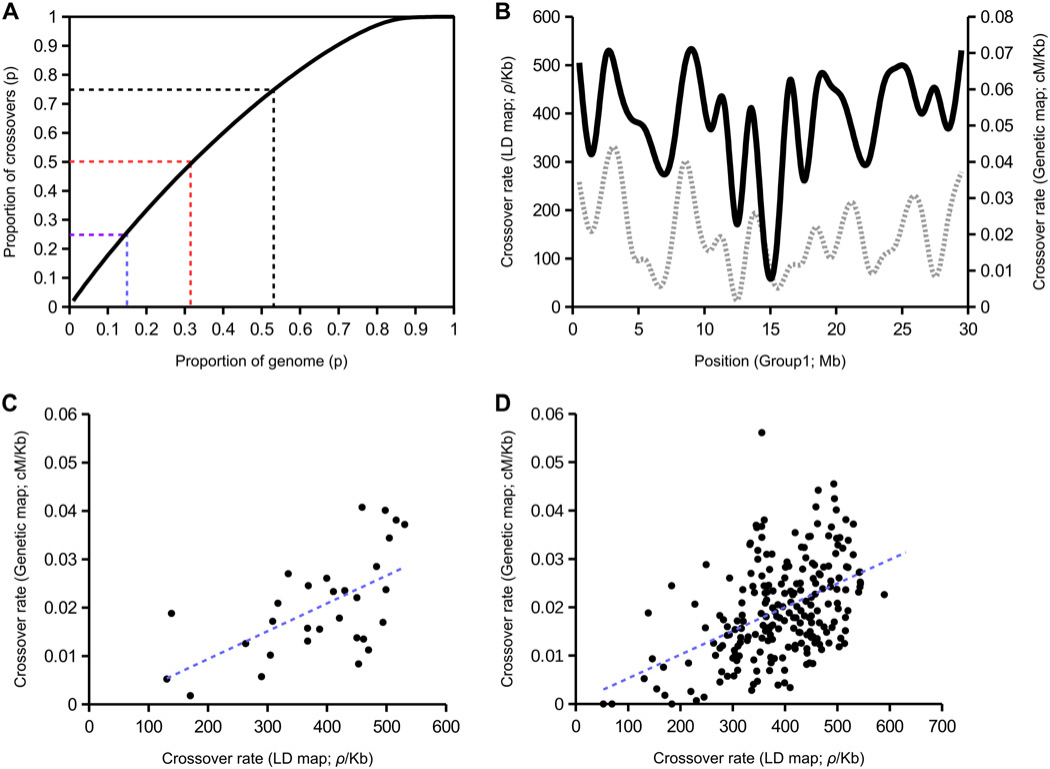
Population recombination rates inferred from linkage disequilibrium among SNPs using LDHAT in comparison with a genetic map [3]. (A) Proportion of recombination as a function of the proportion of genome in which it occurs. (B) Variation in recombination rates along the large metacentric chromosome Group1 (LD map: solid black line; genetic map from [3]: grey dashed line). The maps are transformed to the same resolution of 1 Mb windows. (C) Correlation between recombination rates in the LD-based map and genetic map from [3] (R^2^ = 0.341; p = 0.000424) across chromosome Group1 in windows of 1 Mb. (D) Correspondence between the two maps across the whole genome in windows of 1 Mb (R^2^ = 0.213; p<10^-5^).

Recombination does not appear to be strongly restricted to a limited portion of the genome (Fig 1A), suggesting that there are not strong hotspots in honeybee genome but a relatively continual recombination landscape. For example 50% of the recombination events in the genome occur in 32% of the genome. In humans, a similar map from population scale sequencing suggests that 50% occurs in less than 10% of sequence [59]. There is however considerable large-scale variation in recombination rates along the chromosomes (Fig 1B shows variation along Group 1). The mean population-scaled recombination rate computed from 100 kb windows is 385 *ρ*/kb, with a standard deviation of 167 *ρ*/kb (see S1 Fig for LD maps of all chromosomes). We find that the LD map is broadly congruent with a previously constructed genetic map [3](e.g. R^2^ = 0.341 for the large metacentric chromosome Group1; Fig 1B and 1C), but the strength of correlations varies among regions and chromosomes (R^2^ = 0.213 across the genome; Figs 1D and S1).

### A strong correlation between genetic variation and local rate of crossing over suggests a pervasive impact of selection

**Fig 2:**
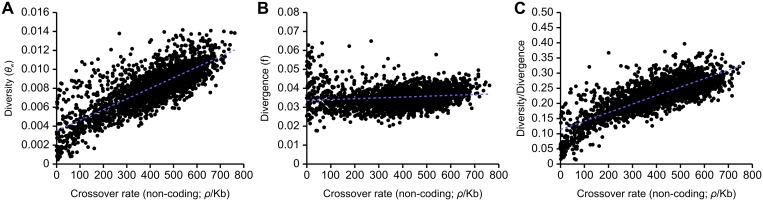
Correlations between estimates of non-coding genetic diversity and divergence with population recombination rates inferred with LDHAT. (A) Correlation between recombination and diversity (R^2^ = 0.615, p<10^-5^). Linear regression: *f*(*x*) = (1.118×10^-5^)*x* + (3.517×10^-3^). (B) Correlation between recombination and divergence between *A*. *mellifera* and *A*. *cerana* (R^2^ = 0.0185, p<10^-5^). Linear regression: *f*(*x*) = (4.439×10^-6^)*x* + (3.360×10^-2^). (C) Correlation between recombination and diversity/divergence (R^2^ = 0.617, p<10^-5^). Linear regression: *f*(*x*) = (2.801×10^-4^)*x* + (1.140×10^-1^). 100 kb genomic windows are used in each comparison.

There is a highly significant correlation between levels of neutral genetic diversity, measured by Watterson’s theta, *θ*_w_, estimated using noncoding sites, and rates of crossing over in the honeybee genome (R^2^ = 0.615, Fig 2A). We also examined the relationship between crossing over rates and divergence between *A*. *mellifera* and *A*. *cerana* and found a significant but very weak correlation (R^2^ = 0.018, Fig 2B). The strong correlation between recombination and genetic variation remains after correcting for divergence (R^2^ = 0.617, Fig 2C). These correlations are also found separately in intronic, intergenic and coding regions (S2 Fig). A highly significant but weaker correlation between diversity/divergence is found using average pairwise heterozygosity (*π*) to measure genetic diversity instead of *θ*_w_ (R^2^ = 0.480). A correlation between genetic variation corrected for divergence and recombination rate is consistent with the pervasive influence of linked selection on patterns of variation, due to background selection, recurrent selective sweeps, or both [28].

It is also possible that fixation biases due to GC-biased gene conversion (gBGC) could contribute to the correlation between genetic diversity and crossover rate, as the strength of gBGC is expected to covary with recombination [42]. To examine this possibility, we recomputed diversity (using *θ*_w_) while removing large classes of non-coding *A*. *mellifera* SNPs and substitutions between *A*. *mellifera* and *A*. *cerana* that may putatively be affected by gBGC. We first removed all variants that change GC content and found diversity to still be positively correlated with crossover rate (R^2^ = 0.563). After observing gBGC among transitions in particular (see below), we next removed all transitions and also observed a positive correlation between diversity and recombination (R^2^ = 0.586). These patterns favour linked selection as a major force in shaping variation in the genome. These correlations are only slightly weaker than the correlations observed when the dataset is randomly subsampled to the same size (R^2^ = 0.573 and R^2^ = 0.594 respectively), which suggests that gBGC has at most a small effect on determining the magnitude of genetic variation in a genomic region.

Average Tajima’s *D* is negative (-1.178) reflecting of skew towards rare variants, as already observed in this African honeybee population [37], which is indicative of population expansion. Tajima’s *D* (measured in 100 kb windows) shows a weak negative correlation with both GC content (R^2^ = 0.114) and recombination rate (R^2^ = 0.015), which indicates a slightly higher skew towards rare variants in regions of high recombination. Pervasive linked selection is expected to generate a skew towards rare variants in regions of low recombination [32], which we do not observe. This could indicate the action of additional factors.

In order to assess whether the association between genetic diversity and inferred recombination rates could be an artefact of having more power to detect recombination in regions of high genetic variation, or due to other biases, we estimated LD-based maps of recombination using datasets where SNPs were removed or using different parameter as follows: i) we produced a dataset where genetic variation (*θ*_w_) in each 100 kb window was capped at 0.002, effectively subsampling data in 98% of 100 kb windows; ii) we produced a dataset where rare variants (minor allele frequency<0.1) were removed; iii) we evaluated the effect of increase the block penalty to 10, which affects the probability of changes in recombination rate between genomic regions. In each case, the resulting LD maps were strongly correlated with the original map (S3 Fig). The strong correlation between levels of genetic variation (in the original dataset) and inferred rates of crossing over remained in the LD maps produced using different parameters (S3 Fig) and are similar to that observed in the original dataset. We therefore conclude that variation in genetic variation across the honeybee genome does not generate biases in inference of recombination and that the correlation between recombination and genetic variation is real.

Levels of genetic variation are reduced close to genes, indicative of an effect of linked selection [37]. In order to determine the effects of linked selection acting on coding sequence on our observed correlation between genetic variation and recombination, we analysed this correlation restricting the analysis to sites at different distances from genes. We find that the correlation between diversity and recombination in intergenic regions is weaker when restricted to sites far from coding sequences. At sites <20 kb from coding sequences R^2^ is 0.364, whereas it is 0.281 at 50–60 kb and only 0.208 at 100–110 kb away (randomly sampling the same amount of data in each case). This supports the interpretation that this correlation is due to the effect of linked selection, as sites under selection are expected to be rarer far from genes. This is also supported by a finding of an excess of SNPs with high *F*_*ST*_ in within coding sequences [37].

### Crossover rates are correlated with GC and CpG content

The honeybee genome has low GC content (on average 34%) but GC content is variable across the genome [26]. To further understand the basis for this variation, and how it relates to recombination rate variation, we first partitioned the genome according to annotations and calculated the average GC content among genes and gene elements. We find that coding, intergenic and intronic regions each have characteristic GC content (S4 Fig). Coding regions are biased toward high GC content (39%), whereas intronic regions are particularly low (23%) and intergenic regions are intermediate (31%). Interestingly, 5’ UTRs have higher on average GC content than 3’ UTRs (31% cf. 24%).

A unique feature of the honeybee genome is an overall excess of CpG dinucleotides (measured by CpG_O/E_), compared to expectations based on frequencies of single bases. CpG_O/E_ is also highly variable between different functional regions of the genome. It is high in noncoding regions (~1.7 in both introns and intergenic regions). However, notably, the coding part of the genome has an average CpG_O/E_ close to the expected (1.04). This is consistent with the observation that methylation in honeybees occurs predominantly in gene bodies [60]. As reported previously [61], CpG_O/E_ is bimodally distributed among genes (S4 Fig). We assigned genes into high or low CpG_O/E_ categories compared to the mean of 1.19. Genes with low average CpG_O/E_ (LCpG; <1.19 CpG_O/E_) have high levels of germline methylation at CpG sites and tend to be associated with cellular housekeeping functions, whereas genes in the higher average CpG_O/E_ class (HCpG; >1.19 CpG_O/E_) have low levels of germline methylation and tend to be caste and tissue specific [37,60–62].

**Fig 3:**
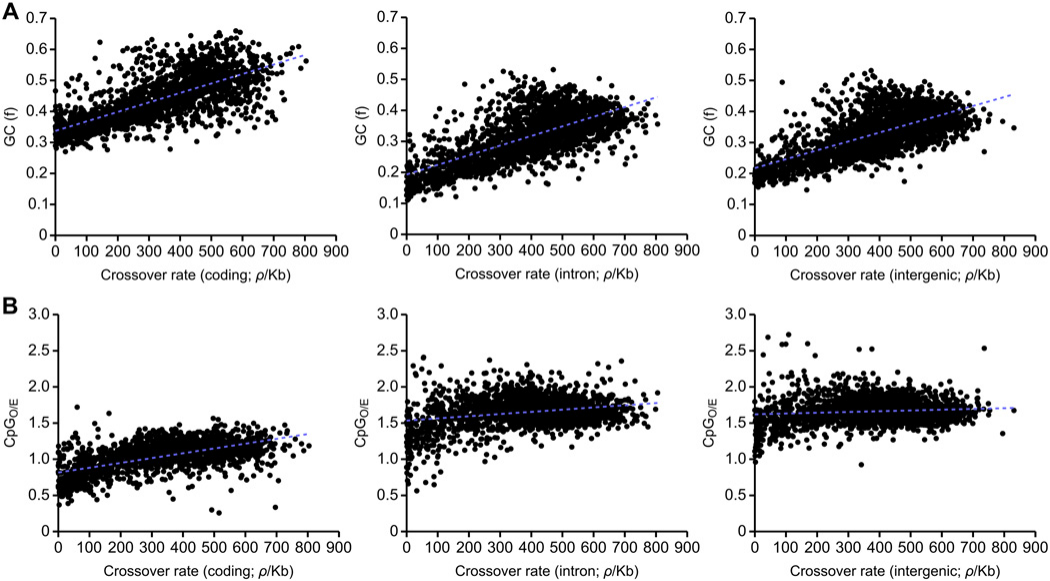
Correlations between recombination rate and GC and CpG content divided according to functional categories. (A) Correlations between recombination rate and GC content in coding (left panel; R^2^ = 0.506, p<10^-5^), intronic regions (centre panel; R^2^ = 0.463, p<10^-5^) and intergenic regions (right panel; R^2^ = 0.446, p<10^-5^). (B) Correlations between recombination rate and CpG_O/E_ in coding (left panel; R^2^ = 0.369, p<10^-5^), intronic regions (centre panel; R^2^ = 0.066, p<10^-5^) and intergenic regions (right panel; R^2^ = 0.011, p = 0.00037). 100 kb genomic windows are used in each comparison.

We find a strong correlation between crossover rates and GC content in the honeybee genome (R^2^ = 0.436). Strong correlations are also observed between GC content and crossover rates within coding (R^2^ = 0.506), intronic (R^2^ = 0.463) and intergenic regions (R^2^ = 0.446; Fig 3A). These correlations are also observed in 5’ and 3’ UTRs (S5 Fig). Such, correlations between GC content and crossover rates are observed in a wide variety of taxa, and could suggest that recombination drives GC content via the process of gBGC [42]. We find that CpG_O/E_ is correlated with recombination in coding sequence (R^2^ = 0.369) but only very weakly correlated in intronic (R^2^ = 0.066) and intergenic regions (R^2^ = 0.011; Fig 3B). Methylation is mainly restricted to coding sequence in honeybees and variation in CpG_O/E_ in coding sequence is likely to reflect differences in germline methylation [60–62]. Conversely, variation in CpG_O/E_ in other parts of the genome is not influenced by DNA methylation and also does not correlate with recombination rate. These results may therefore suggest a role of germline DNA methylation in attenuating recombination rates in the honeybee genome. Interestingly, we also detect this correlation in 3’ UTRs (R^2^ = 0.289), but not in 5’ UTRs, (R^2^ = 0.025; S5 Fig) which could indicate an effect of differential levels of methylation. In support of this, the CpG_O/E_ distribution of 3’ UTRs is shifted towards lower CpG_O/E_ compared to noncoding regions, indicative of higher levels of DNA methylation (S4 Fig; [61]).

### Germline methylated genes have low rates of crossing over

**Fig 4:**
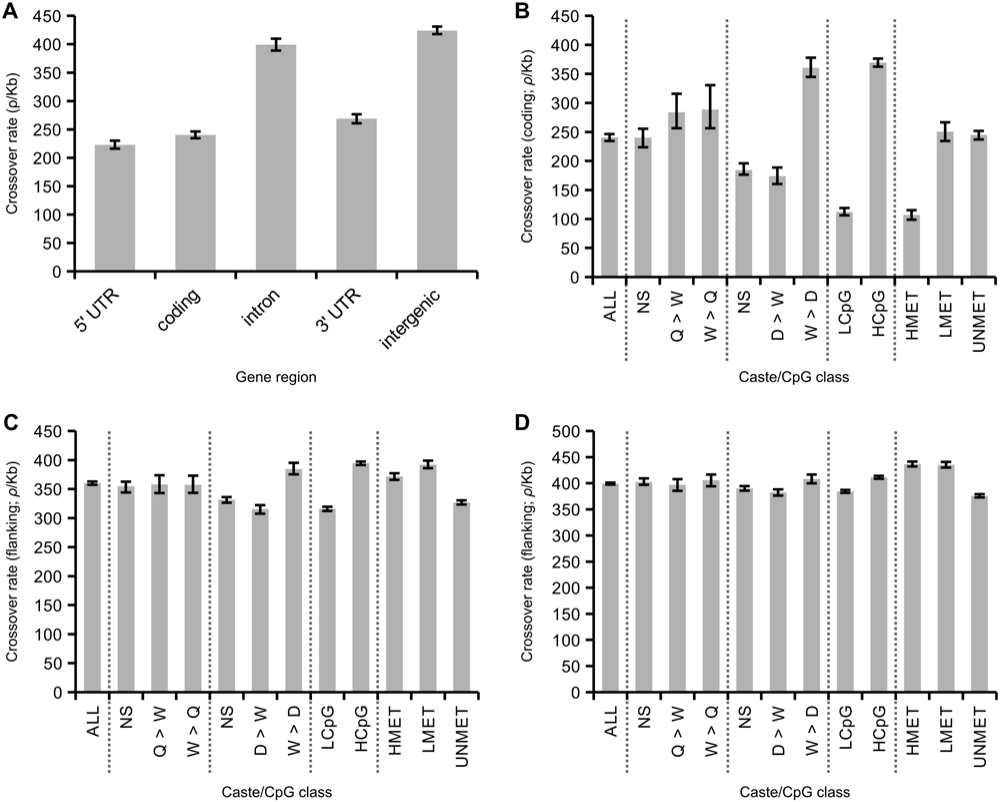
Associations between caste biased gene expression and inferred methylation patterns in honeybees. (A) Variation in average recombination among gene regions. Recombination is significantly reduced in 5’ and 3’ UTRs and coding regions compared with intronic and intergenic regions. (B) Average recombination rate in coding sequence of all genes (left; ~13,000 genes) and from genes divided according to gene expression patterns and CpG content. Two expression datasets are used. One identifies elevated expression in queens compared to workers (Q>W), workers compared to queens (W>Q) or not significantly different between these two castes (NS). The other that identifies elevated expression in drones compared to workers (D>W), workers compared to drones (W>D) or not significantly different between these two castes (NS). Genes are categorised as either low CpG_O/E_ (LCpG) or high CpG_O/E_ (HCpG) in their coding sequence. Genes are also divided according to experimentally measured levels of germline methylation: highly methylated (HMET), lowly methylated (LMET) and unmethylated (UNMET). (C) Recombination rates in noncoding regions flanking genes. Rates are inferred from noncoding regions of 50 kb starting at 10 kb away from either side of a gene to reduce the influence asserted by genic recombination properties. 95% confidence intervals generated from 200 bootstrap subsamples of all genes attributed to a particular gene class. (D) The same as C but using windows of 100 kb starting at 50 kb from each gene.

Average rates of crossing over are reduced in coding sequence and UTRs compared to noncoding regions (Fig 4A). This suggests the presence of specific factors that reduce recombination specifically within genes. We next examined how variation in patterns of gene expression and inferred levels of germline methylation are associated with crossover rate in genes (Fig 4B). Previous studies have suggested that genes with worker-biased expression tend to have high recombination rates [4,39]. To test this, we first compared rates of crossing over within genes with biased expression in queens compared to workers and vice versa [63]. There were no significant differences in crossover rates between these gene categories (p = 0.61, bootstrap test), although the caste-biased genes had higher than average crossover rates (18% increase; p<0.01; average for coding regions = 240 *ρ*/kb). We next compared crossover rates in genes with biased expression in drones compared to workers and vice versa [64]. Here we found highly elevated recombination rates in worker-biased genes (50% increase compared to average; p<0.01) and decreased recombination rates in drone-biased genes (28% decrease; p<0.01) and unbiased genes (23% decrease; p<0.01). We conclude that worker-biased genes have higher recombination rates compared to drone-biased genes, but not compared to queen-biased genes. These results suggest that genes with elevated expression in both female castes (queens and workers) tend to have higher recombination rates, rather than those specifically expressed in workers.

We used two measures to estimate the potential association between levels of germline methylation and rates of crossing over in genes: 1) levels of CpG_O/E_ in coding sequences and 2) estimates based on direct detection of methylated CpG sites in sperm and egg using bisulphite sequencing [62]. Genes were classified as HCpG and LCpG based high or low values of CpG_O/E_ as described earlier. These two measures are highly correlated. We detect significant methylation in 39% of the LCpG genes and 14% of the HCpG genes. Out of all genes where we detect methylation, 60% of the CpGs are methylated in the coding sequence of LCpG genes compared to only 18% in the HCpG genes (S6 Fig). We classified genes as HMET, LMET or UNMET based on the observation of high, low or undetected levels of methylation in the germline. The HMET category had significantly lower average CpG_O/E_ compared to other categories. However, the UNMET class has a bimodal distribution of CpG_O/E_, where 33% of genes have values of CpG_O/E_ <0.7, which could potentially represent germline-methylated genes that were not detected experimentally.

The average crossover rate among LCpG genes is only 29% of the rate esimated in HCpG genes (p<0.01), consistent with an effect of germline methylation suppressing recombination, particularly in HCpG genes (Fig 4B). Inferred levels of methylation are strongly correlated with patterns of gene expression: female-biased genes tend to be HCpG and highly recombining, whereas male-biased genes tend to be LCpG and have lower recombination rates. These patterns also correlate with levels of genetic variation: LCpG genes have on average 45% lower genetic diversity than HCpG genes [37]. The association between levels of recombination and experimentally inferred levels of germline methylation is consistent with these results. Highly methylated genes have low levels of crossing over, similar to those observed in LCpG genes.

A potential concern is that estimates of *ρ* made by LDHAT are affected by local variation in *N*_*E*_ across the genome, which could lead to underestimation of recombination rate in regions of low genetic variation. Since *ρ* and *θ* are correlated, we conducted additional high resolution scans to test whether the differences in *ρ* we observe in coding relative to intergenic regions and in LCpG genes relative to HCpG genes could be due biases in inference caused by differences in local genetic diversity between these regions [37]. We measured *ρ* and *θ* in 1 kb windows across the genome. We found that *ρ* is consistently higher outside of genes than inside of genes at given levels of *θ* (S7 Fig). Likewise, HCpG coding sequences are typically associated with higher *ρ* than LCpG coding sequences at given levels of *θ* (S7 Fig), although the difference is less clear in regions where diversity is very high. We conclude that our inference of *ρ* in these regions detect significantly different crossover rates that are not merely mirroring local levels of genetic diversity.

We next aimed to test whether the associations between caste biased gene expression, CpG levels, and crossover rates were indicative of specific gene categories being preferentially located in regions of certain recombination rates, or whether the association was restricted to recombination in coding sequences. Such a regional effect would be predicted if there was a selective advantage for worker-biased genes to occur in regions of high recombination [39]. We therefore compared patterns of gene expression and methylation to crossover rates in gene-flanking sequence, using 50 kb regions located 10–60 kb from each side of the genes (Fig 4C) and in 100 kb regions located 50–150 kb from each side of the genes (Fig 4D).

As expected, crossover rates increase with increasing distance from the gene (average rate at 10–60 kb distance = 360 *ρ*/kb; average rate at 50–150 kb distance = 399 *ρ*/kb). In addition the associations between expression patterns and CpG levels are greatly reduced. The average decrease in crossover rates of drone-biased genes in flanking regions at 10 kb distance is only 13% compared to all genes and 4% of average >50 kb away. Crossover rates in the queen vs. worker comparisons are indistinguishable from the average rates in both >10 kb and >50 kb. The differences in crossover rates between LCpG and HCpG genes are also reduced in flanking regions compared to crossover rates within coding sequence. There is an 3.37x difference in crossover rates between LCpG and HCpG within coding sequence (p<0.01) but this is reduced to 1.24x and 1.07x in the >10 kb and >50 kb flanking regions respectively. Associations between methylation classes and crossover rate are also significantly weakened in flanking sequence. These results indicate that crossover rates vary greatly between genes and correlate with both patterns of gene expression and levels of germline methylation. However, the finding that these associations are restricted to crossover rates in coding regions is indicative of a direct effect of these factors rather than an accumulation of certain types of genes in regions of high or low recombination, which would be predicted if there was an evolutionary advantage of worker genes being located in regions of high recombination [39].

We tested whether the associations between crossover rates and gene expression and CpG content were independent of each other. Genes that are biased in workers compared to drones are enriched in the HCpG class, so it is not clear which of these two factors is driving the association with high recombination rates. We therefore subdivided both datasets of caste-biased genes according to HCpG and LCpG classes. We found that the large differences in crossover rates in HCpG and LCpG remain irrespective of patterns of gene expression (S8 Fig): for the same gene expression class, crossover rates are 2.3–3.4x higher in HCpG compared to LCpG genes. However, within each CpG class, the difference in crossover rates between drone and worker biased genes is smaller (1.3x higher in HCpG genes and 1.8x higher in LCpG genes). Hence, variation in CpG content is the strongest predictor of recombination rate in our dataset. One interpretation for this finding is that variation in levels of germline methylation is the strongest factor determining variation in recombination rates within genes in the honeybee genome. However, the associations we observe with crossover rates and gene expression patterns cannot be completely explained as an effect of differences in inferred levels of germline methylation.

### GC-biased gene conversion dominates patterns of genetic variation

**Fig 5:**
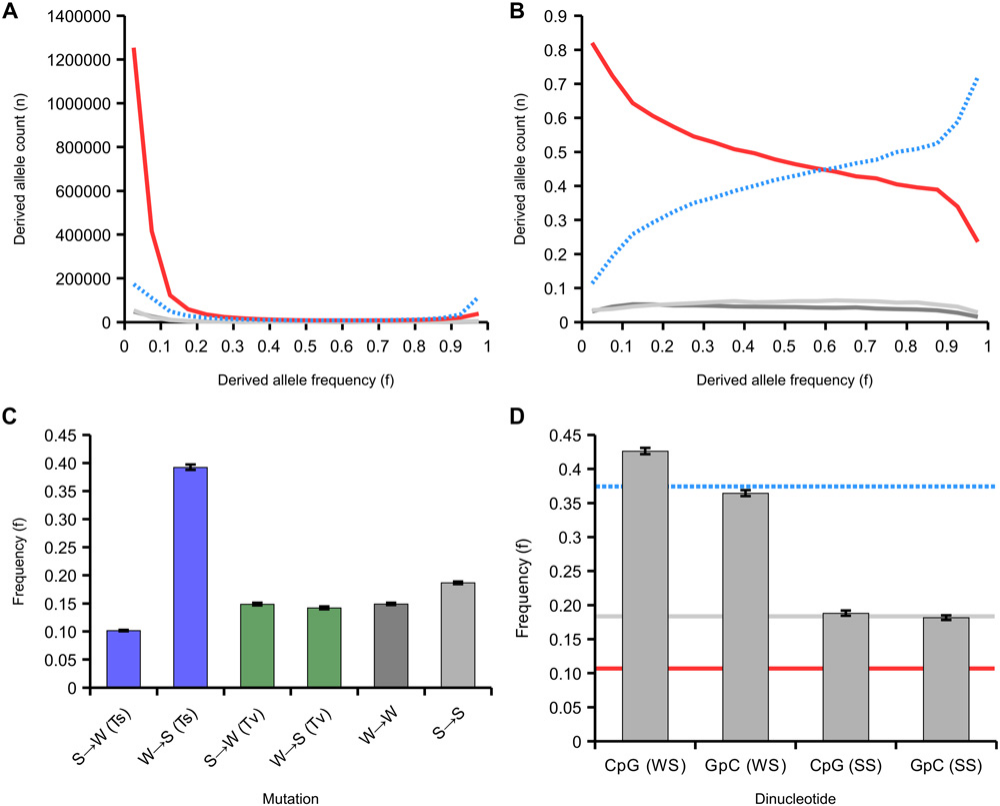
Site frequency spectrum of derived variants. (A) The site frequency spectra of 2,983,700 variants classified according to their mutational origin as defined by comparison to the outgroup. Variants are: SW (strong-to-weak; red line; 2,037,148 SNPs in total); WS (weak-to-strong; blue dashed line; 719,365 SNPs); SS (strong-to-strong; light grey; 117,806 SNPs); WW (weak-to-weak; dark grey; 109,381 SNPs). (B) The relative site frequency spectra of the four variants (variants and colours as in A). (C) Average frequencies of derived variants of different mutational classes. SW and WS mutations are classified as transitions (Ti, blue) or transversions (Tv, green), SS and WW as in A. (D) Average frequency of WS and SS variants that create CpG sites compared to those that create GpC sites. The blue dashed line represents the mean frequency of WS variants, the red line is the mean frequency of SW variants and the grey line is the mean frequency of SS variants. 95% confidence intervals generated from 200 bootstrap subsamples of all SNPs attributed to a particular class.

The site frequency spectrum in our dataset is dominated by low frequency AT alleles, which make up 80% of the rare variants (allele frequency<10%) across all SNPs, but only 51% of common variants (allele frequency 40–50%), a highly significant difference (p<10^-5^, Fisher’s exact test; S9 Fig). By comparing homologous genomic regions between *A*. *mellifera* and *A*. *cerana*, we were able to infer the probabilities that either allele represented the ancestral or derived state at 2,983,700 SNPs using a weighted parsimony method (see Methods). We categorised each allele at a SNP as weak (A or T) or strong (G or C). At strong-to-weak (SW) SNPs, the S allele is ancestral and the W allele is derived, whereas weak-to-strong (WS) SNPs are defined as the reverse. The derived allele frequency spectrum consists mostly of strong-to-weak (SW) mutations (2,037,148 SNPs), and these are strongly biased towards occurring at low frequencies (Fig 5A). Weak-to-strong (WS) mutations are fewer overall (719,365 SNPs), but are shifted toward high frequency or nearly fixed. Analysis of the proportions of variants of each type across the allele frequency spectrum therefore reveals a decline in SW and increase in WS variants with increasing allele frequency. WS variants make up 15% of variants at allele frequencies <0.1 but 79% of variants at allele frequencies >0.9 (Fig 5B).

This highly skewed site frequency spectrum is indicative of a strongly AT-biased pattern of mutation coupled with a fixation bias towards WS mutations. Such a fixation bias could be generated by a strong effect of GC-biased gene conversion (gBGC), which manifests as a bias towards transmission of GC alleles. In order to further investigate this process, we quantified the average allele frequencies of a variety of classes of variants. We found that WS transitions segregate on average at 3.6x higher allele frequency in the population than SW transitions (p<0.01; Fig 5C). However, the average frequencies of WS and SW transversions were similar to each other (14.1% and 14.9%, respectively) and close to the average derived allele frequency in the sample (16.8%). These results are consistent with a fixation bias driven by WS transitions (A→G or T→C), which could indicate that gBGC specifically targets transitions in the honeybee genome. A potential mechanism for this could be that heteroduplex mismatches between two alleles formed by a transition are repaired with a greater GC-bias than other mismatches in honeybees during meiosis. To our knowledge, such a mechanism has not been observed in any other species.

We next tested whether gBGC could potentially be responsible for the huge excess of CpG dinucleotides observed in the honeybee genome. CpG sites are highly enriched in the genome (CpG_O/E_ = 1.64) but GpC occur at numbers close to the expected (GpC_O/E_ = 1.03). This suggests an excess number of WS mutations that generate CpG sites occur or that they have a greater chance of fixation. We detect significantly elevated average frequencies of CpG-generating WS variants (0.43) compared with GpC-generating WS variants (0.37) in the population, although there is no difference between CpG and GpC generating SS variants, which are not expected to be affected by gBGC (Fig 5D). The proportion of WS variants that generate CpG sites compared with those that generate GpC sites is 1.17 at low derived allele frequencies (<0.1) but 1.72 at high derived allele frequencies (>0.9; p<10^-5^; S10 Fig). Conversely, the proportion of SW variants at ancestral CpG sites compared with those that are ancestrally GpC is 1.73 at low derived allele frequencies (<0.1) and 1.30 at high derived allele frequencies (>0.9; p<10^-5^). Hence, there appears to be a fixation bias in favour of CpG-creating mutations and against CpG-destroying ones. These results could explain the excess of CpGs in the honeybee genome. This suggests that fixation bias due to gBGC displays neighbour-dependency in honeybees, which has not been reported for any other species. In addition to gBGC, it is also possible that WS CpG-creating mutations could be positively selected if CpG were selectively maintained.

**Fig 6:**
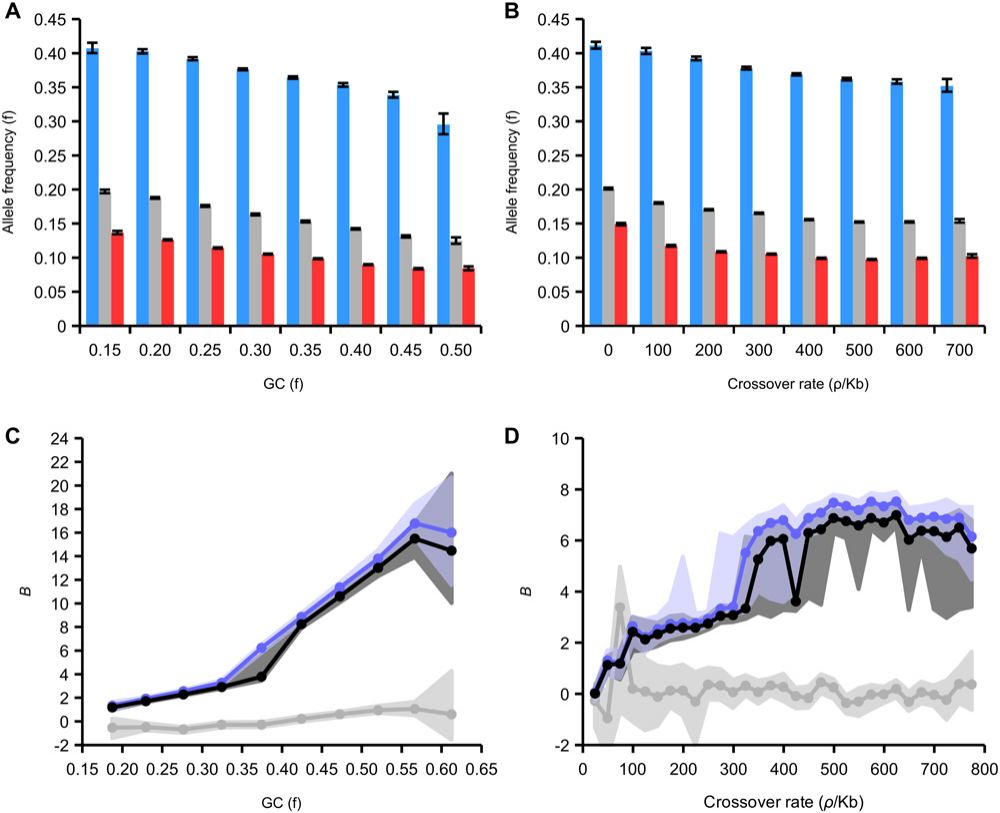
Effect of GC content and recombination on derived allele frequencies in intergenic regions. (A) Average allele frequency of all, WS, SW variants relative to GC content. 95% confidence intervals generated from 200 bootstrap subsamples of all SNPs attributed to a particular GC bin. (B) Average allele frequency of all, WS, SW variants relative to recombination. 95% confidence intervals generated from 200 bootstrap subsamples of all SNPs attributed to a particular rate bin. (C) Maximum likelihood estimation of *B*, the gBGC coefficient, derived from the site frequency spectrum plotted against GC content (black line, all variants; blue line, transitions; grey line, transversions). (D) Maximum likelihood estimates of *B* relative to recombination rate (variants and colours as in C).

We sought to investigate the dependency of the fixation bias due to gBGC on GC content and recombination rate. WS variants occur at higher frequency on average than SW variants in all GC and recombination rate categories (Fig 6A and 6B). The difference between these frequencies increases as a function of both of these variables. For example, the average frequency of SW variants is reduced by 56% in regions of high GC (0.50–0.55) compared to low GC (0.15–0.20) but the average frequency of WS variants is only reduced by 25%. This indicates that the site frequency spectrum is more skewed towards high frequency WS alleles in regions of high recombination and GC content. This trend suggests that the strength of gBGC is stronger in regions of high recombination and GC content.

We estimated the strength of the transmission bias due to gBGC in the honeybee genome using the model of Glémin et al. [57]. This method estimates the population-scaled gBGC parameter *B*, which is equivalent to 3*N*_*E*_ *b*, where *b* is the transmission bias in favour of GC alleles. This method allows taking into account both polarization errors of mutations, which can lead spurious or biased signature of gBGC, and demographic effects distorting site frequency spectra (see Methods). The maximum-likelihood estimates of *B* reveal a strong influence of gBGC on the fixation process of alleles. Average *B* in the genome is 5.71, which is 15 times higher than average levels of *B* estimated from the site frequency spectrum in human populations (0.38)[57]. Levels of *B* this high are only found in the most extreme regions of the human genome that likely correspond to recombination hotspots [57]. Estimates of B vary between transitions and transversions (*B* in transitions 6.47; *B* in transversion 0.03). These estimates are consistent with our earlier inference that the effects of gBGC are restricted to transitions in honeybee.

We estimated *B* in subsets of the genome divided according to GC content and recombination rates (Fig 5C and 5D; S1 and S2 Tables). The association with GC content is strongest, and *B* increases from around 2 in the lowest GC content category (<0.2) to a maximum of >15 in GC content >0.55. The estimates of *B* increase from <1 in in the regions of lowest recombination to a maximum of around 7 in regions with crossover rates >400 *ρ*/kb. Even the lowest values of *B* are several times higher than the average in humans and some parts of the honeybee genome have extreme values of B. We expected *B* to be correlated with crossover rate, as gBGC is a recombination-associated process. However, here we find a stronger correlation with GC content. One reason for this could be that GC content is a more accurate indicator of recombination rates than our LD-based map because it is the result of the action of gBGC over evolutionary time. Another possibility is that our LD-based map predominantly measures crossover rates, which may not be strongly correlated with non-crossover rates. It is also possible that gBGC has a stronger correlation with non-crossover rates than crossover rates, as observed in humans [47].

The method also allows us to estimate the AT mutational bias, λ. We estimate the average bias over the whole dataset as 11.69. The strong AT mutational bias appeared specific to transitions (λ in transitions 13.09; λ in transversion 3.03). Estimates of λ vary slightly across the genome. They are inferred to be higher (9.71–12.40) in regions of lower recombination (<300 *ρ*/kb) and lower (7.91—9.94) in regions of higher recombination (>300 *ρ*/kb; S2 Table). When fitting the population genetics model we estimated high polarization error probabilities for WS mutations (between 10 and 20%). We therefore performed simulations to estimate the potential effects of this on our estimates of *B*. We find that the high levels of gBGC may explain the high SNP polarization errors and that our estimates of *B* are robust to these errors (S1 Text). Moreover, the high and significant skewness of the folded GC spectra (see S1 and S2 Tables), which are not sensitive to polarization errors, is congruent with a force pushing GC content far from the expected mutational equilibrium.

## Discussion

Here we used patterns of linkage disequilibrium in 30 diploid honeybee genomes to estimate variation in crossover rates across each chromosome. Our results are consistent with previous results suggesting that meiotic homologous recombination occurs at extreme rates in honeybees [2–4]. The landscape of recombination rate variation in the honeybee genome does not appear highly punctate as found in a wide rage of other species [7–10]. We find a strong correlation between genetic variation and crossover rates indicative of pervasive influence of linked selection. Our results are also informative about the structure and organisation of the genome in relation to intensity of recombination, and identify specific factors that are likely to mediate recombination rates. We also show that recombination has extensive influence over population genetics and genome evolution in honeybees via the process of GC-biased gene conversion (gBGC), which results in a bias in favour of fixation of WS mutations [42]. The strength of the bias in honeybees is an order of magnitude higher than previously observed in other species.

Although our estimate of average crossover rate of 26 cM/Mb is similar to previous estimates, the correlation with a previous genetic map [3] is only moderate. It is possible that differences in the genome assemblies used by the studies contributed to these differences—our study used Amel_4.5, whereas the Solignac et al. [3] study used Amel_4.0. It is also possible that additional factors that affect patterns of LD, such as selection and gBGC, could affect our estimates of recombination rates. Temporal variation in recombination rates could also explain the moderate correlation between the two maps. The map by Solignac et al. [3] is based on markers segregating in the progeny of two queens thus corresponding to present-day recombination rates, whereas our map integrates all recombination events over a historical period. Another possibility is that there is variation between individuals in recombination landscape. In particular, we have focussed on samples from African honeybee subspecies, whereas the map by Solignac et al. [3] used two queens of European origin. In the future it would be interesting to investigate the genetic determinants of any inter-individual variation in recombination rates.

Studies of fine-scale variation in recombination rate have revealed large variation in rates across the genome in a wide range of sexual eukaryotes [7–10]. These include plants [8], fungi [46], and vertebrates [7]. Conversely, invertebrates such as the nematode worm *Caenorhabditis elegans* [19] and the fruit fly *D*. *melanogaster* do not seem to have strong hotspots [20–23]. Interestingly, recombination does not seem to be required for synapsis in these species [65,66], and mechanisms that are not dependent on sequence features may govern location of crossover events. The distribution of recombination events in honeybees also seems to follow this pattern. This suggests that a PRDM9-like protein that targets specific motifs during initiation of recombination is not present in honeybees further supports the notion that PRDM9 is a derived state in vertebrates [8].

The reasons for extremely high recombination rates in honeybees and other social insects are elusive. One possibility is high recombination rates are connected to the evolution of worker phenotypes, because the evolution of sociality specifically involved positive selection on worker behaviour [39]. This could potentially favour increased recombination rate in the vicinity genes involved in worker phenotypes because selection is more efficient in regions of high recombination [67]. Alternatively, high variability in worker phenotypes could be needed to maintain a stable and diverse workforce, which could also potentially favour increased recombination rates in the vicinity of genes involved in worker phenotypes [40,41]. A possible prediction of both of these scenarios is that genes with biased expression in the worker caste are preferentially located in highly recombining regions of the genome.

Previous analyses of honeybee recombination found increased levels in worker genes, consistent with the above hypotheses [4,39]. However, here we report that a) elevated recombination rates are observed in genes with biased expression in either of the female castes and not specifically in worker-biased genes, b) pattern of gene expression are not well correlated with recombination rates in noncoding flanking regions, and c) germline methylation patterns inferred by CpG_O/E_ are more strongly associated with recombination rates than gene expression patterns are. Our data are consistent with a model where germline gene body methylation is the main modulator of recombination rate in genes and that correlations with gene expression are a side effect of this. Although evolution of eusociality likely involved strong selection for high recombination rates there is no evidence so far to indicate that it involved increases in recombination rate in specific genomic regions.

Both housekeeping genes, and genes mainly expressed in drones, are inferred to be germline methylated and have suppressed levels of crossing over [37,61]. Genes with high CpG_O/E_ have crossover rates similar to intergenic regions. These observations are consistent with the view that DNA methylation is the main cause of reduction of recombination rates in genes and variation in recombination rates between genes, although we cannot rule out the effect of another factor indirectly associated with methylation. It is important to note that the association between recombination and CpG_O/E_ in the honeybee genes could also be influenced by gBGC, which generates new CpG sites [68]. However, the link between CpG_O/E_ and methylation in honeybee genes is well established [60,61] and confirmed in this study. Methylation is generally restricted to gene bodies in honeybees (75% of methylated CpGs are found in exons [60]) and recombination rates in noncoding regions are higher outside of genes. Methylation has been suggested to suppress recombination rate in a variety of species including the fungus *Ascobolus* [69] and angiosperm *Arabidopsis* [70]. Vertebrate genomes tend to be highly methylated, but hypomethylated CpG islands have elevated recombination rates in some species [18,71,72]. We therefore hypothesise that germline DNA methylation suppresses recombination in honeybees.

We find a strong correlation between recombination rate and levels of neutral genetic variation that remains after correcting for mutation rate inferred from levels of divergence with an outgroup, *A*. *cerana*. Similar correlations are observed in a diverse range of species and are believed to reflect the effects of recurrent selective sweeps (positive selection) and/or the effects of selection removing linked deleterious variants (background selection) [28,32]. If selection occurs at similar rates across the genome, then it will have a greater effect on linked variation in regions of low recombination leading to this general correlation. The predicted effect of selection depends on the rate at which it occurs across the genome and whether variants are strongly or weakly selected. Interestingly, we also find that the correlation between diversity and crossover rates is weaker in regions far from genes, which is consistent with a lower density of functional sites and hence potential targets of selection in these regions. Our findings are therefore consistent with a pervasive impact of selection on genome variation in honeybees similar to inferences in fruit flies [32]. Recombination increases the efficacy of selection [29,67,73] and high levels of selection (e.g. due to recurrent selective sweeps) are a potential explanation for the extreme recombination rates observed in honeybees.

Our analysis also indicates a dominant effect of gBGC on genetic variation in honeybees. The derived allele frequency spectrum contains a large excess of SW mutations segregating at low frequencies, and an excess of WS mutations at high frequencies close to fixation. This skewed site frequency spectrum is indicative of a strongly AT-biased pattern of mutation and a fixation bias towards WS mutations, consistent with a strong effect of gBGC. However, our analysis indicates that gBGC in the honeybee has two features that have not been reported in other species. First, the WS fixation bias appears much stronger for transition than transversion mutations, which could reflect a greater strength of GC-bias in repair of mismatches caused generated by transitions during recombination. Second, we find evidence that this bias is stronger in CpG compared to GpC sites. This suggests that the repair bias could also be neighbour dependent in honeybees. This could explain the massive excess of CpG sites observed in the honeybee genome.

The reasons for these specific biases are unclear. Quantification of gBGC in humans found no evidence for repair bias towards transitions or CpG sites [57] and no such biases have not been observed in other species either. *In vivo* experiments in mitotic mammalian cells suggest that G/T mispairs in DNA, which can be generated by transitions, are strongly biased towards being repaired to GC rather than AT [74,75]. However, these biases result from the base excision repair (BER) pathway, and mismatches during recombination are mainly repaired by mismatch repair (MMR). Our results could therefore indicate a greater role of BER in repair of mismatches during recombination in honeybees, or they could suggest that these biases occur in MMR in honeybees. It has been suggested that such repair biases exist in order to correct common types of mutations, in particular due to hypermutability at methylated CpG sites in mammals [76]. We observe a strong AT mutation bias, particularly in transitions, which is counteracted by a strong GC fixation bias in transitions. Levels of methylation and CpG mutation in honeybee are generally low, but are restricted to genes. Mutations at such sites could be more accurately repaired by the CpG-biased mechanisms we infer here.

We estimate strength of the fixation bias due to gBGC in honeybees to be incredibly high (average *B* = 5.71), and much greater than observed previously in any other species (average *B* in humans is 0.38). Such a high level of gBGC is likely recent because the average GC content of the honeybee genome (0.34) is much lower than the equilibrium GC content predicted by the balance between gBGC and AT mutational bias (GC* = 1/(1+λe^-*B*^) ≈ 0.96). At values of *B* less than one, as observed in the human genome, gBGC is not expected to dominate over random genetic drift [57]. However the values of *B* estimated here are substantially greater than one, indicative of a dominant influence on molecular evolution. Indeed, across much of the genome, they exceed *B* = 8.7, the value estimated for human hotspots, which is expected to result in the fixation of a substantially elevated number of deleterious nucleotide substitutions [54].

The magnitude of *B* depends on both effective population size, *N*_*E*_, and the transmission bias in favour of GC alleles, *b* (*B* = 4*N*_*E*_ *b* for diploids and *B* = 3*N*_*E*_ *b* for haplodiploids). Using estimates of *N*_*E*_ of 10,000 [77] for humans and 500,000 for honeybees [37] leads to estimates of *b* of 9.5 x 10^-6^ and 3.8 x 10^-6^, respectively. Hence, we infer that the transmission bias in humans should be 2.5 times stronger than in bees. However, due to the higher *N*_*E*_ in bees, this lower transmission bias still has an extreme effect on the allele frequency distribution. The honeybee is still unusual in having extremely high levels of gBGC, as related taxa with high *N*_*E*_ do not seem to have similarly high levels. In particular, the site frequency spectrum in *D*. *melanogaster* does not appear strongly skewed [78]. It therefore seems likely that the extreme recombination rates in honeybees are linked to high levels of gBGC, even if the transmission bias in meiosis is not greater in magnitude than humans. In addition, compared to *Drosophila*, it is also possible that the high AT mutation bias in honeybees has selected for a stronger *b* per meiosis. It should also be noted that recombination only occurs in honeybee females, which suggests that the transmission bias in female meiosis is likely to be twice our estimate here, which is a sex-averaged estimate.

The strong skews in site frequency spectrum and fixation biases are incompatible with a standard model of population genetics whereby the fate of alleles is determined by genetic drift and selection. The process of gBGC has a major influence on probability of fixation of an allele in honeybee populations. This has major implications for molecular evolution, as it can interfere with the removal of harmful alleles and fixation of beneficial alleles by natural selection and cause fixation of weakly deleterious mutations. Selection for higher recombination rates in honeybees therefore appears to have entailed the considerable additional cost of strong gBGC.

## Methods

### The LD map

We aimed to produce a high-resolution map of recombination in the Western Honeybee *Apis mellifera* using 30 diploid sequences from African worker bees collected in South Africa and Nigeria. Although these populations are geographically separated, analyses of population structure suggest that this sample can be regarded as panmictic and a single population. The bees were sequenced as part of a different study and short read mapping, genotype calling, filtering and phasing procedures are described in Wallberg et al. [37].

Watterson’s estimator [79] was used to calculate the population mutation rate per base (*θ*_w_) as a measure of genetic diversity across the genome. Diversity, GC content and CpG_O/E_ was calculated in windows of 100 kb along the chromosomes using the current reference genome (Amel_4.5; [80]). These statistics were averaged across the full window and for each type of functional element (coding, intron, UTRs and intergenic sequence; coordinates according to the recent gene annotations in OGSv3.2; [80]) in the window. The African population includes 6.2 million single nucleotide polymorphisms (SNPs), corresponding to an average level of genetic diversity of *θ*_w_ = 0.008. The reversible-jump MCMC algorithm *interval* of the LDHAT program [81] was used to estimate the mean population-scaled recombination rate coefficient *ρ* (rho) across regions (or intervals), which in honeybees is taken as *ρ* = 3*N*_*E*_ *r* (3*N*_*E*_ is due to honeybee haplodiploidy) and where *r* is the genetic map distance over a region. The interval method fits a uniform recombination rate over a region from patterns of linkage disequilibrium (LD) among genotypes. The LDHAT recombination map (hereafter referred to as the LD map) was estimated along the chromosomes in segments of up to 2,000 variable sites. The segments were arranged to never span across scaffolds and had an average physical length of 63 kb. For each segment, the interval program was run for 1.1 million iterations and the chain was sampled every 10,000 iterations, following a burn-in of 100,000 iterations. We evaluated the performance of different block penalties (see below). A map inferred with a block penalty of 1 was taken as the canonical LD map for the study.

Levels of genetic diversity are highly variable along the honeybee chromosomes and correlates with functional elements caste biased expression and nucleotide composition [37]. We therefore performed an analysis to determine whether our method could be biased towards detecting high recombination in regions of high SNP density. Three measures were put in place in order to study the effect of local diversity and LD on the inference of broad-scale recombination from our data:

- Reduction of variability in diversity. Within each block of 100 kb, the diversity of every functional element (according to the recent gene annotations in OGSv3.2; [80]) was independently capped at *θ*_w_≈0.002 by randomly subsampling the SNPs, resulting in a thinned dataset spanning 1.5 million SNPs.
- Pruning of rare variants. A substantial fraction of the dataset consists of variants that occur at low frequency in the population. We specifically removed all 4.5 million SNPs with a minor allele frequency (MAF) <10%, reducing the dataset to 1.7 million common variants for an average *θ*_w_≈0.0022. LDHAT was then rerun with the manipulated datasets using the parameters specified above.
- Low and high block penalties. The block penalty parameter is used to control the sensitivity to local changes in LD. The larger the penalty, the more evidence is needed to accept a change in recombination rate and the smoother the map. We applied two block penalty parameter values (1 and 10) to assess the impact of this parameter on the rate estimates.

The LDHAT recombination map (hereafter referred to as the LD map) was compared to the GC and CpG_O/E_ composition computed across the full length of each gene in the OGSv3.2 gene annotation and according to intervals of each type of functional element. Gene lists with accessions associated with biased gene expression between queens and workers [63], as well as between drones and workers [64] were queried in order to further assess the interaction between recombination and caste function. The gene lists were subdivided into classes of low or high CpG_O/E_ in order to facilitate analyses of the influence of both sequence composition and caste function on recombination. The significance of differences in crossover rates between gene expression and low or high CpG_O/E_ categories were measured using a bootstrap test. We randomly resampled 200 pseudo-replicates from each class and compared their values in order to generate confidence intervals and estimate significance.

### Germline methylation data

We estimated levels of germline methylation in genes using data from Drewell et al. [62]. Significantly methylated CpGs (mCpGs) were originally detected using short read bisulfite sequencing of honeybee egg and sperm cells and mapped against v2.0 of the honeybee genome. In order to estimate methylation levels in different genes, we merged the two methylation tracks into a single germ line track and associated the coordinates of the mCpGs with overlapping coding sequences using the matching gene model annotation (OGSv1.1). We next measured methylation levels in two ways for each accession: i) the number of mCpGs per kb of coding sequence (controlling for the length of the gene); and ii) the proportion of CpGs in the coding sequence of a gene that were methylated (controlling for the actual CpGs available to methylate).

We then used BLAST to link OGSv1.1 accessions to the current OGSv3.2 accessions, for which we have estimated CpG_O/E_ and recombination rates. 8901 genes were linked across the two annotation systems and included in the downstream analyses. Out of the 8901 genes, the coding sequence of 2449 genes were found to be methylated in at least one CpG site whereas 6452 genes had no evidence of methylation and were classified as unmethylated (UNMET). We divided the methylated genes into two equally sized low methylation frequency (LMET) and high methylation frequency classes (HMET). We estimated the average crossover rates for these categories (UNMET, LMET, HMET) and generated 95% confidence intervals from 200 bootstrap replicates.

### The genetic map

The LD map was compared to an experimental recombination map (hereafter referred to as the genetic map) produced by Solignac et al. [3] from parent-offspring recombinant frequencies inferred from >2,000 evenly spaced microsatellite markers. The markers and genetic distances of the genetic map had originally been computed for an older version of the genome (Amel_4.0; 183 Mb). In order to facilitate a 1:1 comparison between the two methods, we identified the locations of the corresponding marker coordinates for Amel_4.5 (229 Mb) using BLAST [82] of 2 kb flanking sequence associated with each marker. Out of the 2008 original markers, 1974 markers could be mapped unambiguously to Amel_4.5. The remaining markers were not included due to primer sequences aligning to different scaffolds or chromosomes or at unexpectedly large distances from each other compared to the original positions. Between the two versions of the reference genome, there had been extensive reorganisation and reorientation of scaffolds. Many genetic distances had originally been estimated across scaffolds, which themselves may have been subject to change. By querying multiple 2 kb segments of each of the v4 scaffolds against the v4.5 chromosomes with BLAST, we detected orientation changes in 124 out of 371 scaffolds (33%). These changes often caused previously adjacent markers to be separated by additional markers on the new reference sequence, resulting in overlapping genetic intervals and a much-reduced average recombination rate of 11.3 cM/Mb across the genome, compared to the reported rate of 22 cM/Mb. By including genetic distances stretching across adjacent scaffolds only if they were both plus-oriented, we produced a new genetic map with an average rate to 22.3 cM/Mb, which was next correlated to the LD map in windows of 1 Mb. The last window of each chromosome was only included if it spanned at least 0.5 Mb of sequence.

### Allele frequency spectra and patterns of mutation

The Eastern honeybee *A*. *cerana* is a sister species of *A*. *mellifera* and was used as outgroup in several analyses. Short reads from 10 diploid worker samples were mapped as described in ref. [37] and pooled in order to produce an *A*. *cerana* consensus sequence from sites with a minimum depth of coverage of 5x. The consensus sequence was next used to estimate the nucleotide divergence between the two species and use the outgroup allele to infer the ancestral state at *A*. *mellifera* SNPs. At sites where the ingroup is polymorphic (X|Y) and the outgroup is fixed for one of the two alleles (e.g. X), simple parsimony assumes that the allele shared between the ingroup and the outgroup is the ancestral allele (X) and that a X→Y mutation generated the polymorphism in the ingroup. However, this reconstruction does not take into account the possibility that the other allele (Y) was the true ancestral allele but was substituted in one of the species (Y→X), followed by an Y→X mutation which generated the X|Y polymorphism in the ingroup. To incorporate this uncertainty and reduce the error in the polarization of the mutations, we applied a weighted parsimony method that incorporates substitutions to estimate the conditional probabilities that either allele represent the ancestral or derived state given an ingroup polymorphism and an outgroup allele [49]. The polymorphisms were next classified as transitions (Ti) or transversions (Tv) and whether they were weak-to-strong (WS), strong-to-weak (SW); weak-to-weak (WW) or strong-to-strong (SS), whereby a weak allele is A or T and a strong allele is G or C. In total, 3.02M SNPs were classified according to this scheme. The average population frequency of the derived allele (*f*_D_) of each SNP was estimated across the genome and related to dinucleotide context, regional GC content and recombination rates (computed from windows of 100 kb).

### Estimation of gBGC

We used the method of Glémin et al. [57] to estimate the strength of gBGC. In brief, this method fits a population genetics model to the derived allele frequency (DAF) spectra of the three kinds of mutations, 1) W→S, 2) S→W, and 3) S→S and W→W. This model takes into account the departures from the equilibrium induced by demography, population structure and/or sampling. Despite modelling an explicit demographic scenario, the model includes fuzzy parameters correcting for the distortion of the spectrum compared to the one expected in an equilibrium population, following the approach of Eyre-Walker et al. [83] and as initially implemented for gBGC in Muyle et al.[84]. Importantly, it also corrects for polarization errors of mutations that can bias gBGC estimates [85]. Because, it was proved to be difficult to estimate the heterogeneity of *B* without additional information to constrain the model [57], we only fitted a constant gBGC model (model M1* in [57]). Given the average GC-content, the AT mutational bias can also be estimated. To get the DAF spectra, the numbers of SNPs detected in each mutational class were summed over the frequency spectrum across the whole dataset. Site frequency spectra were also generated according to bins of local GC (100bp window to either side of the SNP) and regional recombination (1000bp window). We estimated *B* for all mutations and for transitions and transversion separately.

## Supporting Information

S1 Fig Recombination maps for the 16 nuclear chromosomes in the honeybee genome. Recombination rates were inferred from linkage disequilibrium among SNPs using LDHAT (black lines = 1Mb window; blue lines 250 kb window; plotted with a spline smoothing function).
(PDF)

S2 Fig Correlations between estimates of genetic diversity and divergence with population recombination rates inferred with LDHAT divided according to functional categories. (A) Correlation between recombination and diversity in coding (left panel; R^2^ = 0.381, p<10^-5^), intron (centre panel; R^2^ = 0.617, p<10^-5^) and intergenic (right panel; R^2^ = 0.531, p<10^-5^) regions, respectively. (B) Correlation between recombination and divergence between *A*. *mellifera* and *A*. *cerana* in coding (left panel; R^2^ = 0.164, p<10^-5^), intron (centre panel; R^2^ = 0.030, p<10^-5^) and intergenic (right panel; R^2^ = 0.0028, p<10^-5^) regions. (C) Correlation between recombination and diversity/divergence in coding (left panel; R^2^ = 0.229, p<10^-5^), intron (centre panel; R^2^ = 0.590, p<10^-5^) and intergenic (right panel; R^2^ = 0.523, p<10^-5^) regions. 100 kb genomic windows are used in each comparison.
(PDF)

S3 Fig Evaluation of data and parameter dependency in the recombination rate inference. (A) Correlations between rates estimated using all data (*θ*_w_ = 0.008; block penalty = 1) and rates using reduced SNP density or high block penalties. From the left: evenly thinned dataset (*θ*_w_≈0.002; R^2^ = 0.790; p<10^-5^; pruned dataset without rare variants at frequencies <0.1 (*θ*_w_≈0.0022; R^2^ = 0.797; p<10^-5^); rates using block penalty = 10 (R^2^ = 0.900; p<10^-5^). (B) Correlations between diversity estimated using all data and rates using reduced SNP density or high block penalties. From the left: evenly thinned dataset (R^2^ = 0.552; p<10^-5^; pruned dataset without rare variants (R^2^ = 0.484; p<10^-5^); rates using block penalty = 10 (R^2^ = 0.607; p<10^-5^). 100 kb genomic windows are used in each comparison.
(PDF)

S4 Fig GC/CpG distribution in different genomic regions computed ~13,000 genes. (A) GC content (proportion of genes according to bins of 0.01 GC; bold black = coding; blue = 5’-UTR; bold green = 3’-UTR; red = intron; dashed grey = intergenic). (B) CpG_O/E_ content (proportion of genes according to bins of 0.05 GpG_O/E_; regions and colours as in A).
(PDF)

S5 Fig Correlations between recombination rate and GC and CpG content in 5’ and 3’ UTRs. (A) Correlations between recombination rate and GC content in 5’-UTRs (R^2^ = 0.371, p<10^-5^) and 3’-UTRs (R^2^ = 0.388, p<10^-5^). (B) Correlations between recombination rate and CpG_O/E_ in 5’-UTRs (R^2^ = 0.025, p<10^-5^) and 3’-UTRs (R^2^ = 0.289, p<10^-5^). 100 kb genomic windows are used in each comparison.
(PDF)

S6 Fig Association between CpG_O/E_ and germline methylation. (A) Histogram of the genes grouped by CpG_O/E_ values associated with each methylation class: unmethlylated (UNMET, red), high methylation (HMET, black) and low methylation (LMET, blue). The HMET distribution strongly deviates from the other classes, centering around low CpG_O/E_ values. (B) Average CpG_O/E_ values for genes within the three methylation classes (UNMET, LMET and HMET). (C) Average levels of methylation, measured in methylated CpGs / kb for HCpG and LCpG genes. (D) Average levels of methylation, measured in the proportion of CpGs that are methylated for HCpG and LCpG genes. 95% confidence intervals for B-D estimated from 200 bootstrap replicates.
(PDF)

S7 Fig Association between local recombination rates and local genetic diversity in coding and intergenic regions. Crossover rates and diversity were measured in 1 kb windows across the genome. Windows spanning >500 bp of intergenic sequence were classified as intergenic regions. Windows spanning >500 bp of coding sequence were classified as coding regions and further subdivided according to the CpG_O/E_ of the coding sequence (LCpG = CpG_O/E_<1.04; HCpG = CpG_O/E_>1.04). (A) Comparison of crossover rates between all coding and intergenic regions at given levels of genetic variation (dashed lines = mean genetic diversity of the region across all genes; shaded area = 95% confidence intervals generated from 200 bootstrap replicates of each interval). (B) Comparison of crossover rates between LCpG and HCpG coding regions at given levels of genetic variation (dashed lines and shaded areas as in A). (C) The subset of the comparisons include the mean levels of genetic diversity of all coding (*θ*_w_ = 0.0038) and LCpG coding regions (*θ*_w_ = 0.0020).
(PDF)

S8 Fig Correction for methylation/CpG and expression residuals. Average recombination rates of genes with caste biased expression (as in Fig 4) subdivided into classes of low (LCpG) or high (HCpG) CpG content. 95% confidence intervals generated from 200 bootstrap subsamples of all genes attributed to a particular gene class.
(PDF)

S9 Fig Folded site frequency spectrum of minor allele variants. (A) The folded site frequency spectra computed from the minor allele frequencies of the 3M variants analysed in Fig 5 without polarizing the mutations using outgroup information. Minor allele variants are: W (A/T alleles; red line); S (G/C alleles; blue dashed line). (B) The relative site frequency spectra of the two minor allele variants (variants and colors as in A). (C) The number of W and S minor allele variants at low (<0.25) and intermediate (>0.25) frequencies, respectively (variants and colours as in A). There is a significant excess of S alleles segregating at intermediate frequencies (p<10^-5^; Fischer's exact test).
(PDF)

S10 Fig CpG/GpC fixation bias. From left to right, the four bars show a) the proportion of WS variants that generate CpG sites compared with those that generate GpC sites at low derived allele frequencies (<0.1); b) the same ratio at high derived allele frequencies (>0.9); c) the proportion of SW variants at ancestral CpG sites compared with ancestral GpC sites at low derived allele frequencies (<0.1); d) the same ratio at high derived allele frequencies (>0.9).
(PDF)

S1 Table Maximum likelihood estimation of transmission bias, *B*, for categories of GC content. (XLSX)

S2 Table Maximum likelihood estimation of transmission bias, *B*, for categories of recombination. (XLSX)

S1 Text Methods to estimate the strength of gBGC and error rates. (PDF)
