# Supplementary figures and images for: Extreme Recombination Frequencies Shape Genome Variation and Evolution in the Honeybee, Apis mellifera
Source: PLoS Genet. 2015 Apr 22;11(4):e1005189. doi: 10.1371/journal.pgen.1005189 (PMC4406589; doi:10.1371/journal.pgen.1005189)

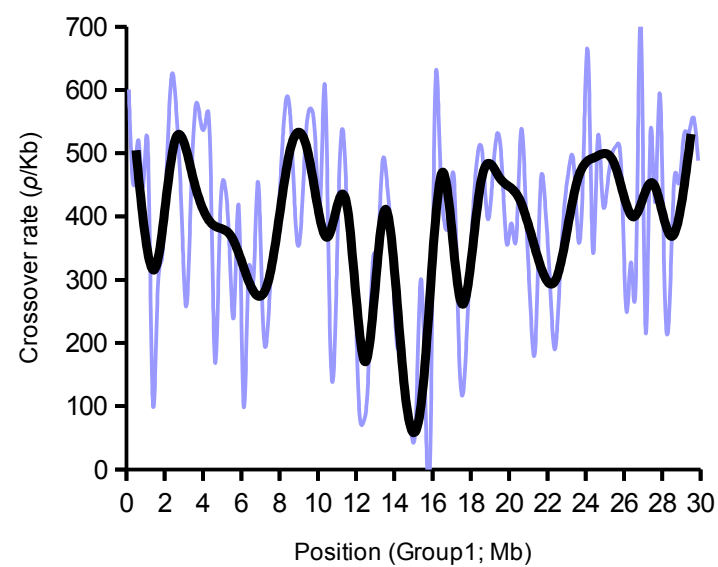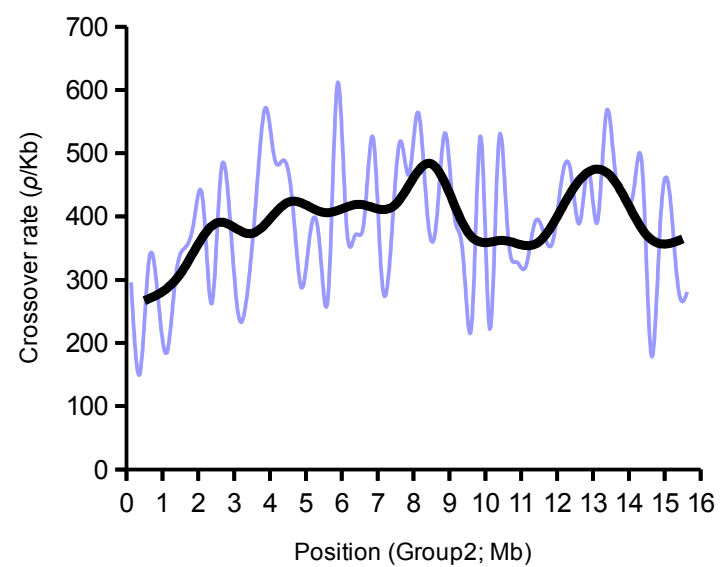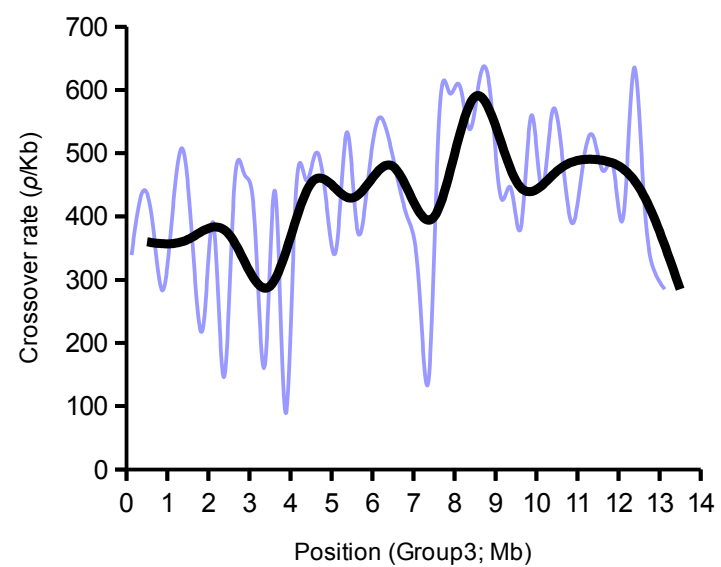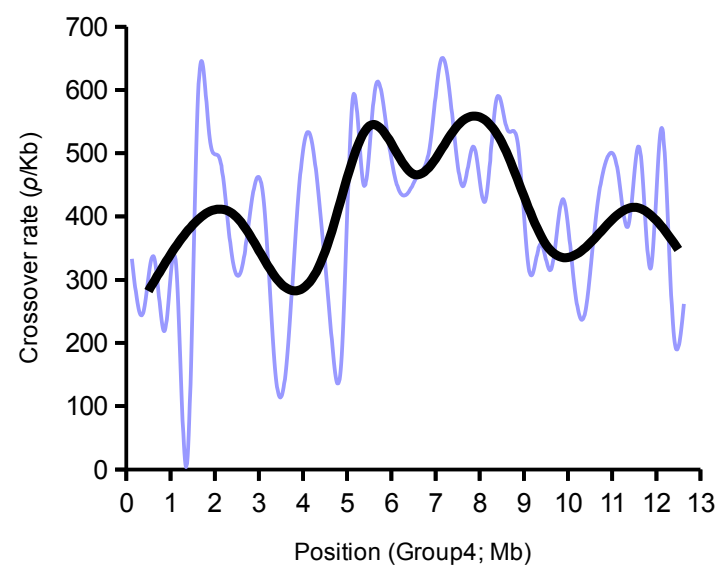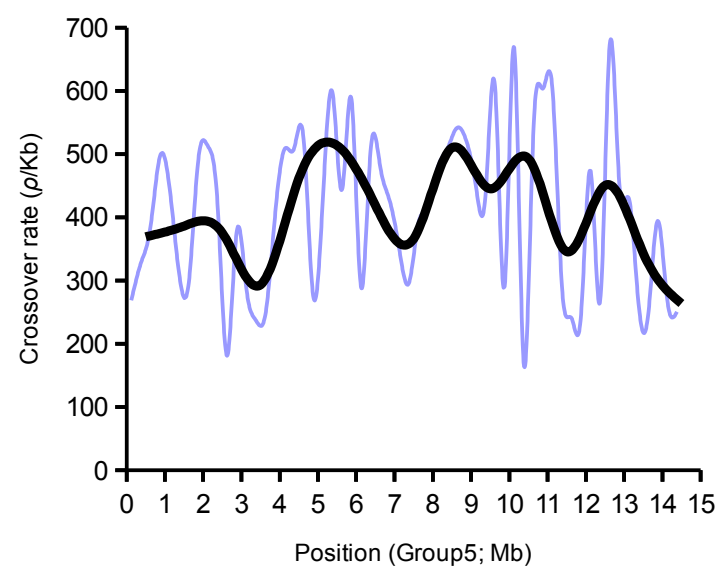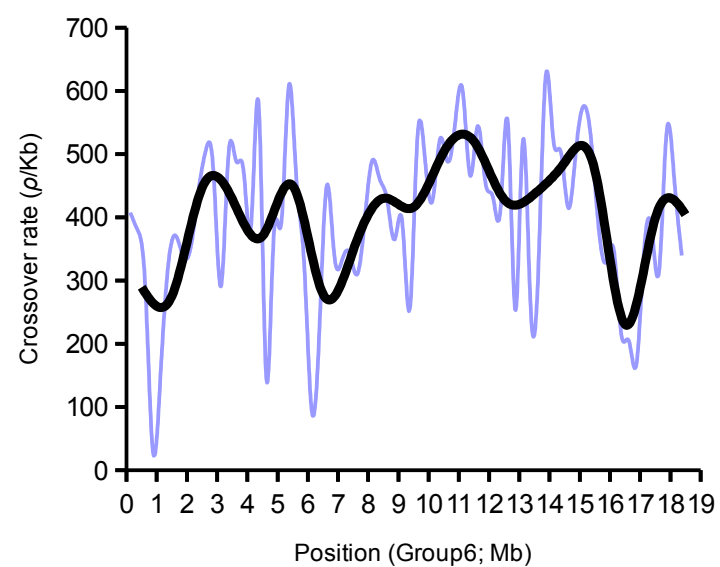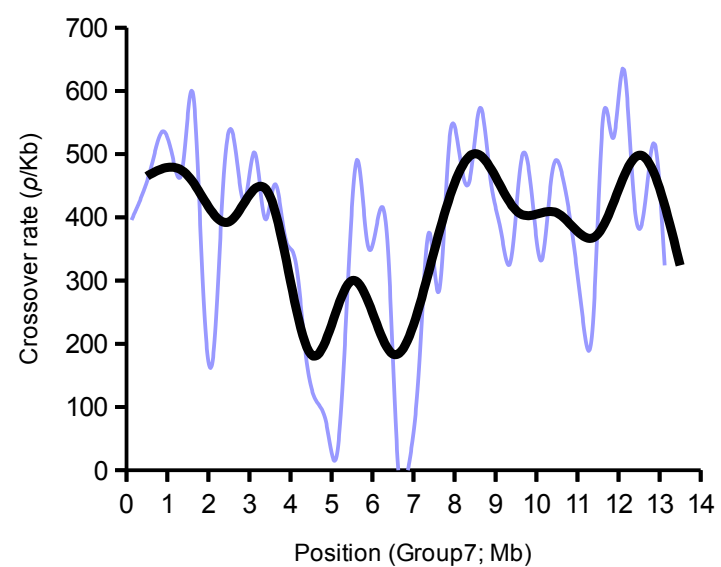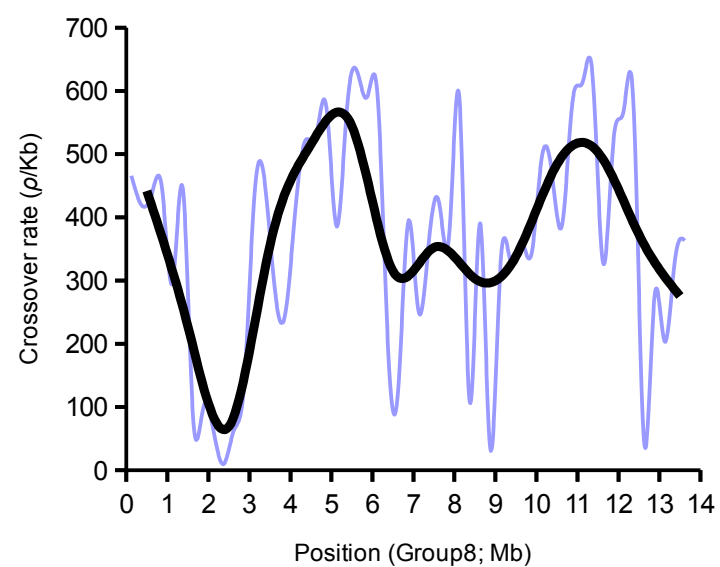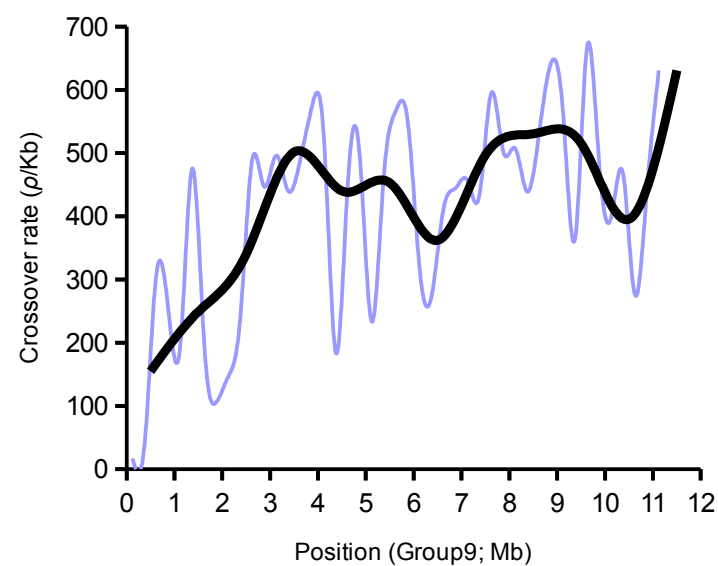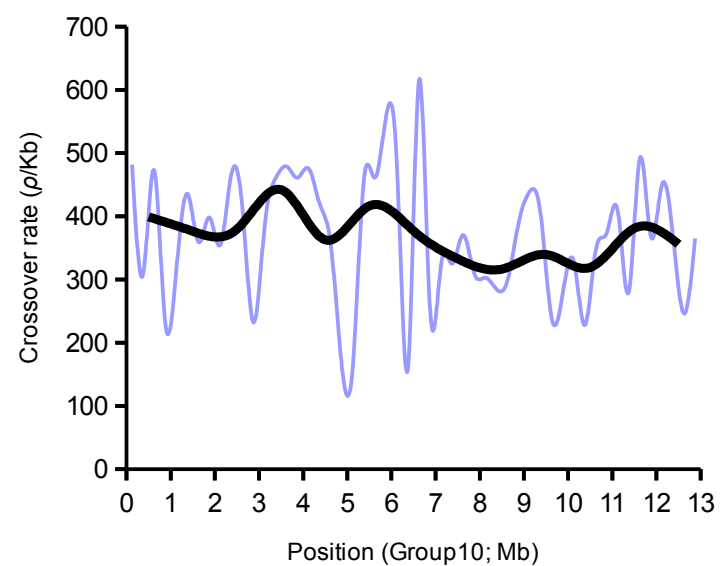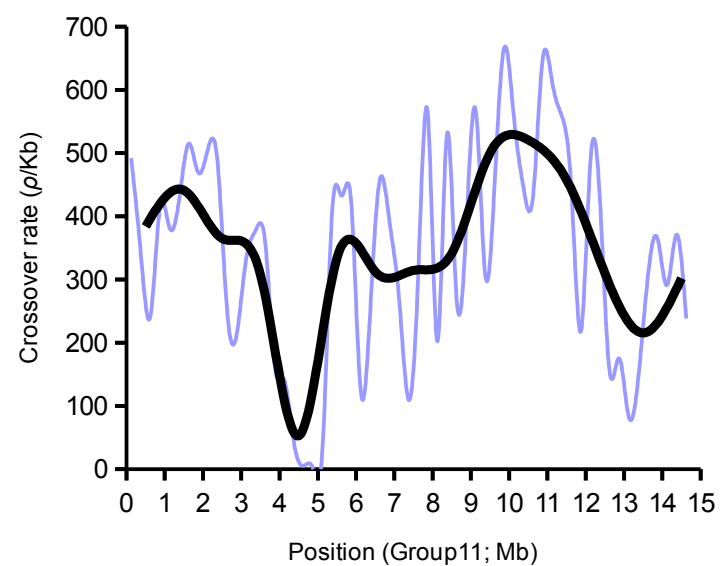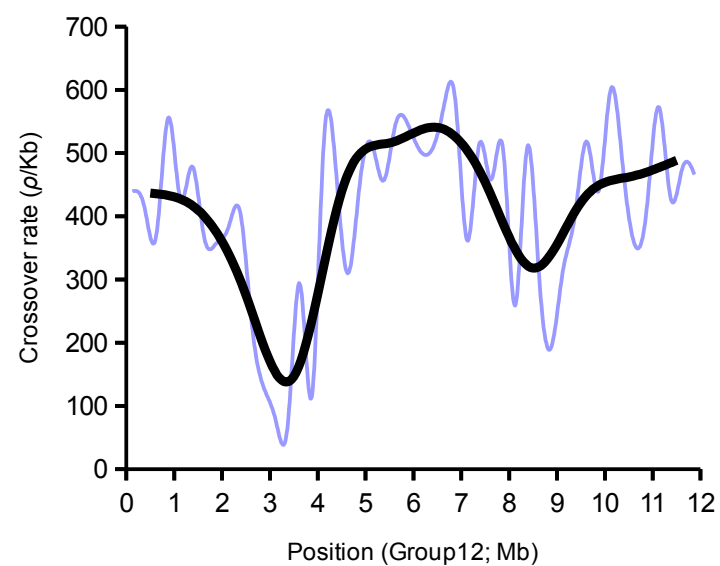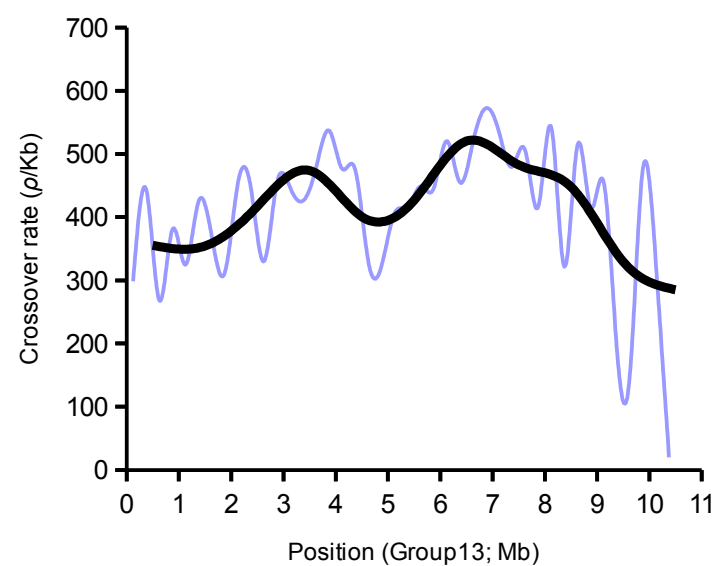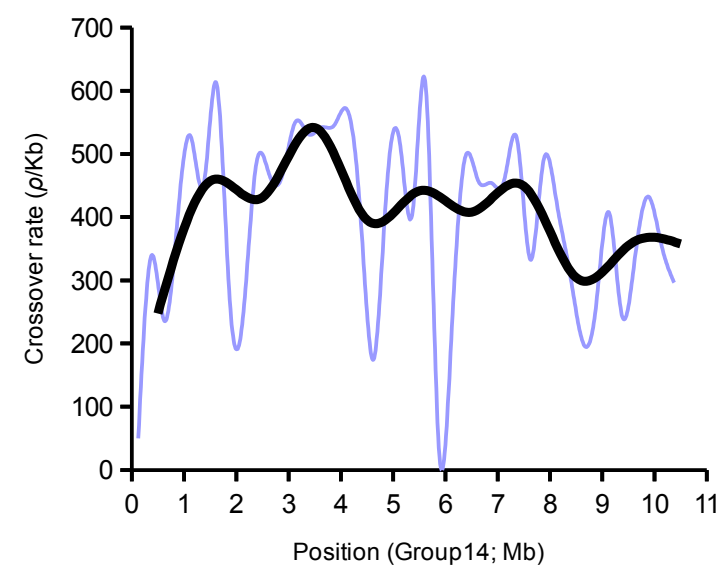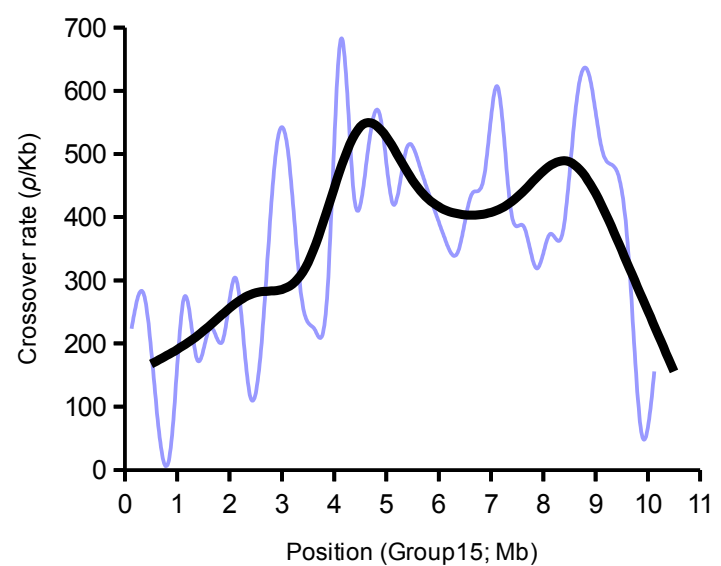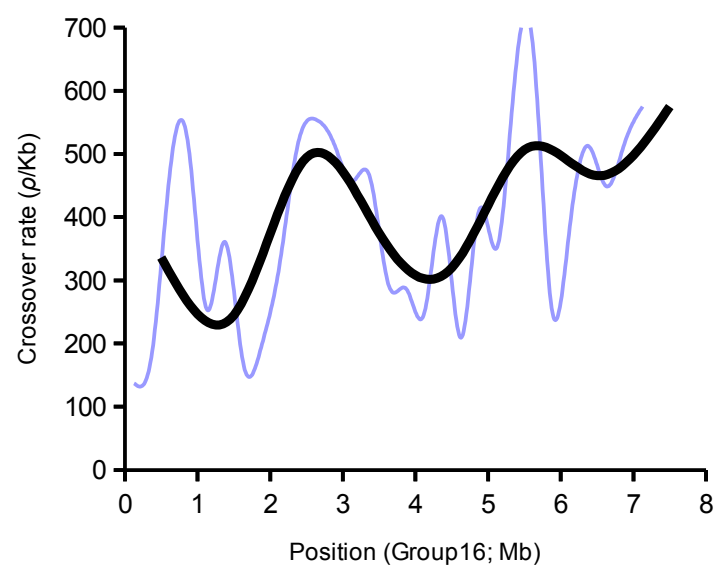

Supplement: S1 Fig — Recombination rates were inferred from linkage disequilibrium among SNPs using LDHAT (black lines = 1Mb window; blue lines 250 kb window; plotted with a spline smoothing function). (PDF) [file pgen.1005189.s001.pdf]

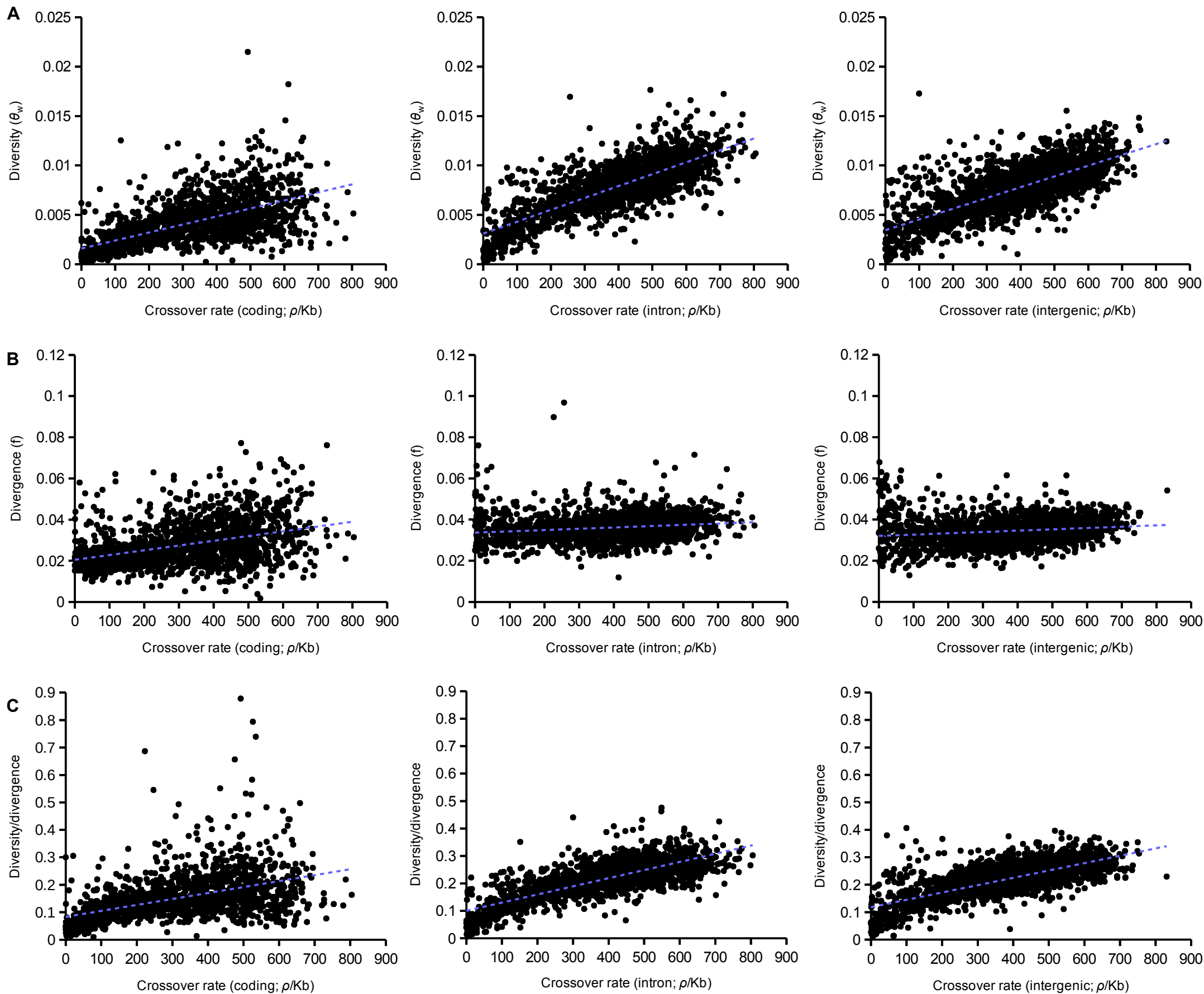

Supplement: S2 Fig — (A) Correlation between recombination and diversity in coding (left panel; R2 = 0.381, p<10-5), intron (centre panel; R2 = 0.617, p<10-5) and intergenic (right panel; R2 = 0.531, p<10-5) regions, respectively. (B) Correlation between recombination and divergence between A. mellifera and A. cerana in coding (left panel; R2 = 0.164, p<10-5), intron (centre panel; R2 = 0.030, p<10-5) and intergenic (right panel; R2 = 0.0028, p<10-5) regions. (C) Correlation between recombination and diversity/divergence in coding (left panel; R2 = 0.229, p<10-5), intron (centre panel; R2 = 0.590, p<10-5) and intergenic (right panel; R2 = 0.523, p<10-5) regions. 100 kb genomic windows are used in each comparison. (PDF) [file pgen.1005189.s002.pdf]

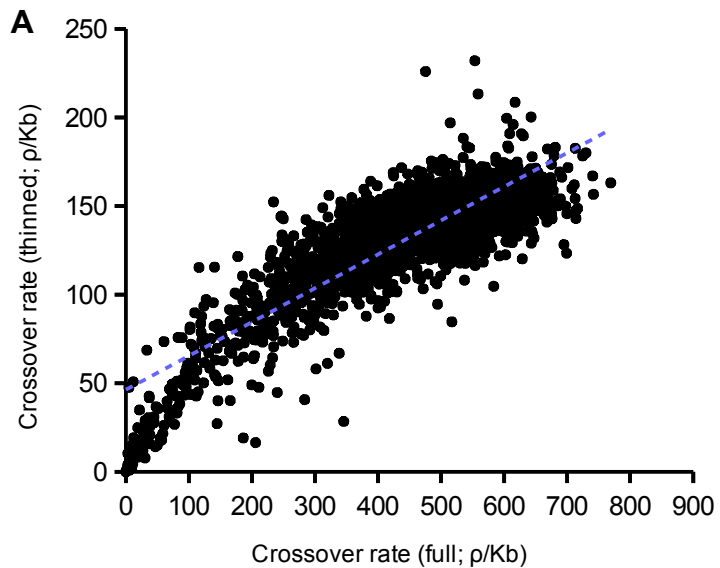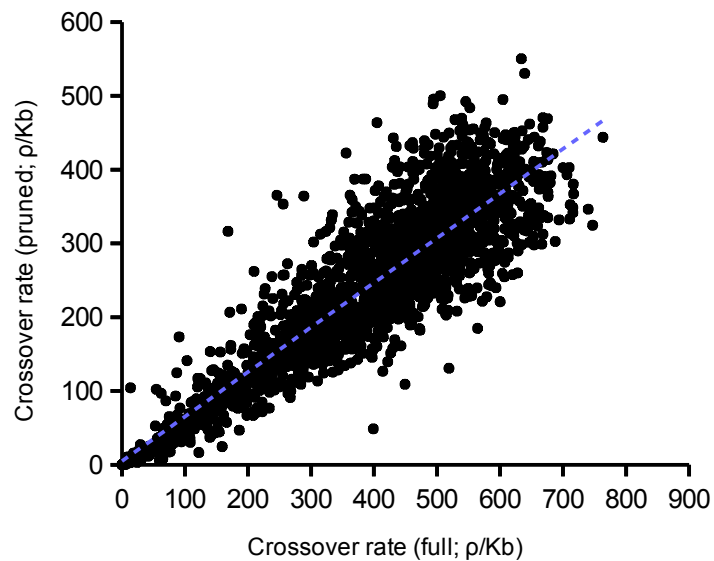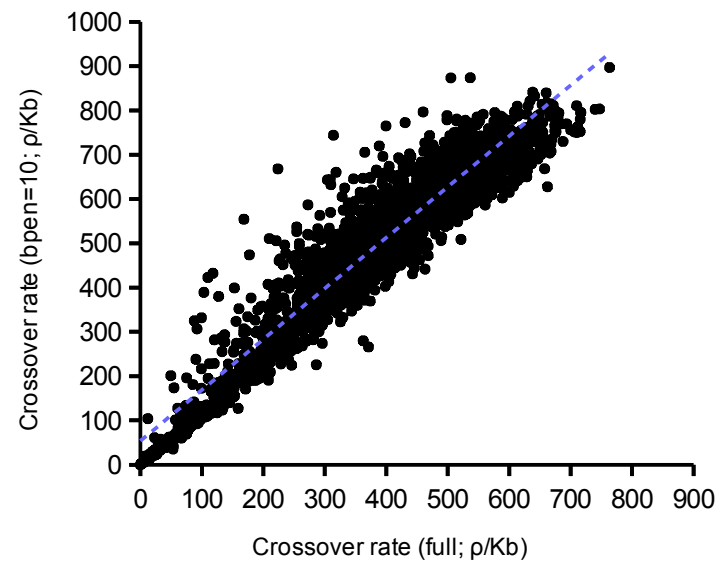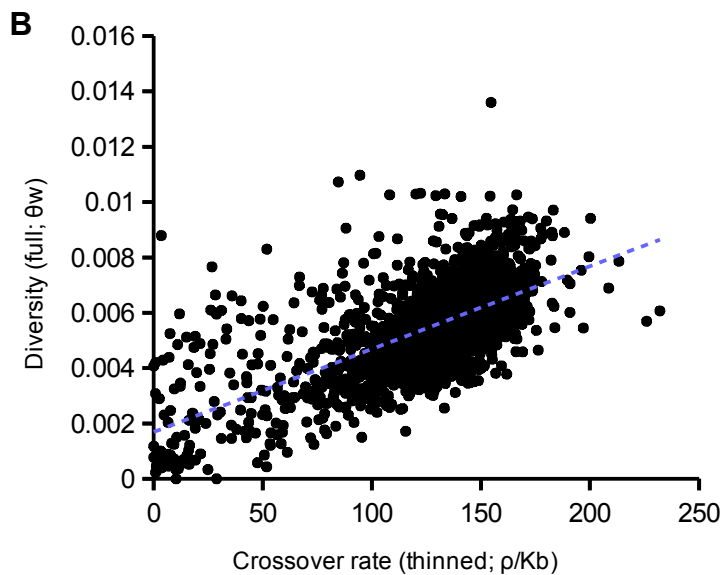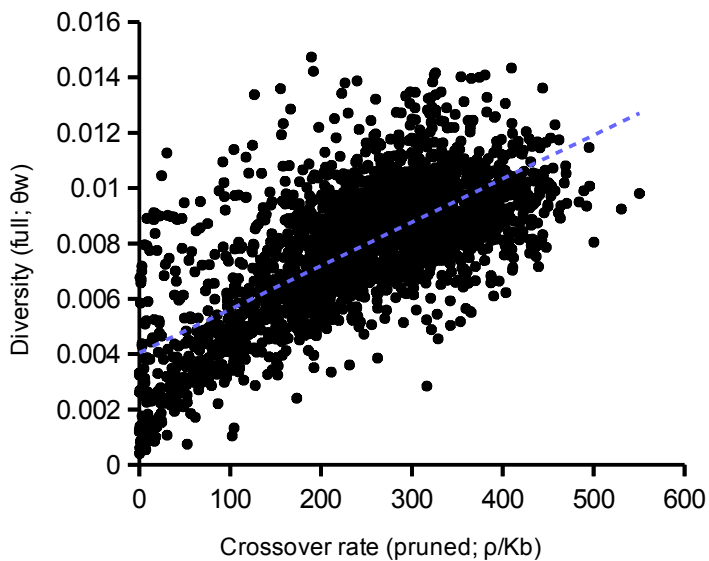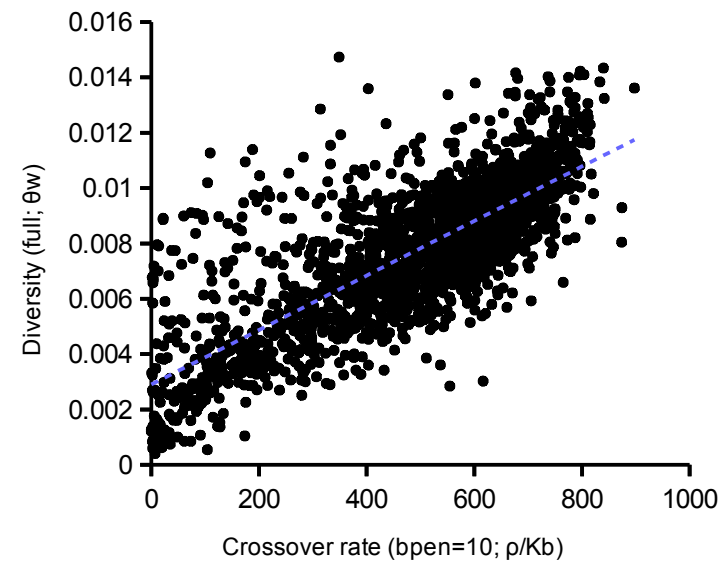

Supplement: S3 Fig — (A) Correlations between rates estimated using all data (θ w = 0.008; block penalty = 1) and rates using reduced SNP density or high block penalties. From the left: evenly thinned dataset (θ w≈0.002; R2 = 0.790; p<10-5; pruned dataset without rare variants at frequencies <0.1 (θ w≈0.0022; R2 = 0.797; p<10-5); rates using block penalty = 10 (R2 = 0.900; p<10-5). (B) Correlations between diversity estimated using all data and rates using reduced SNP density or high block penalties. From the left: evenly thinned dataset (R2 = 0.552; p<10-5; pruned dataset without rare variants (R2 = 0.484; p<10-5); rates using block penalty = 10 (R2 = 0.607; p<10-5). 100 kb genomic windows are used in each comparison. (PDF) [file pgen.1005189.s003.pdf]

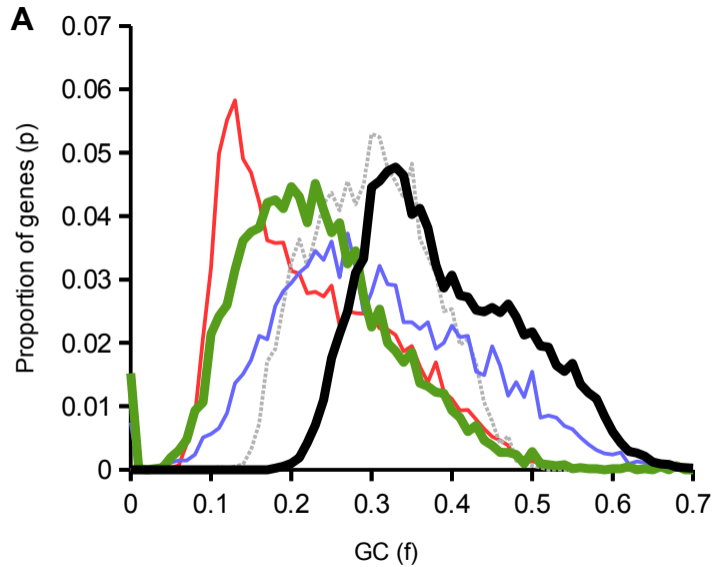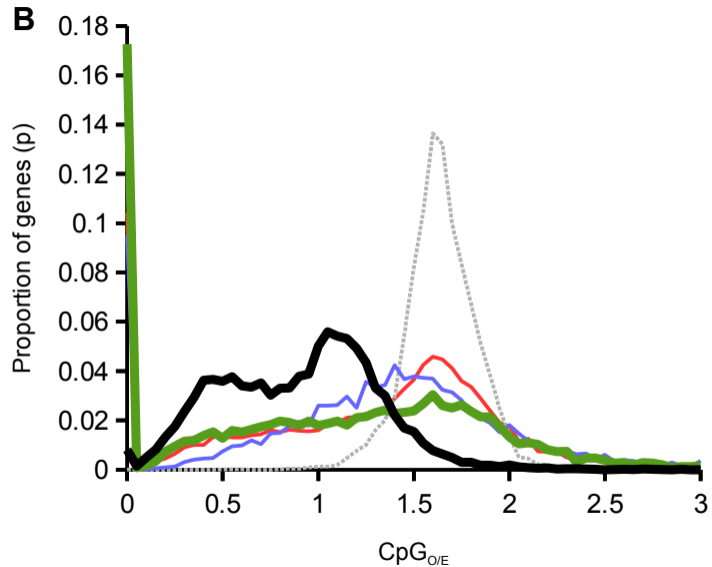

Supplement: S4 Fig — (A) GC content (proportion of genes according to bins of 0.01 GC; bold black = coding; blue = 5’-UTR; bold green = 3’-UTR; red = intron; dashed grey = intergenic). (B) CpGO/E content (proportion of genes according to bins of 0.05 GpGO/E; regions and colours as in A). (PDF) [file pgen.1005189.s004.pdf]

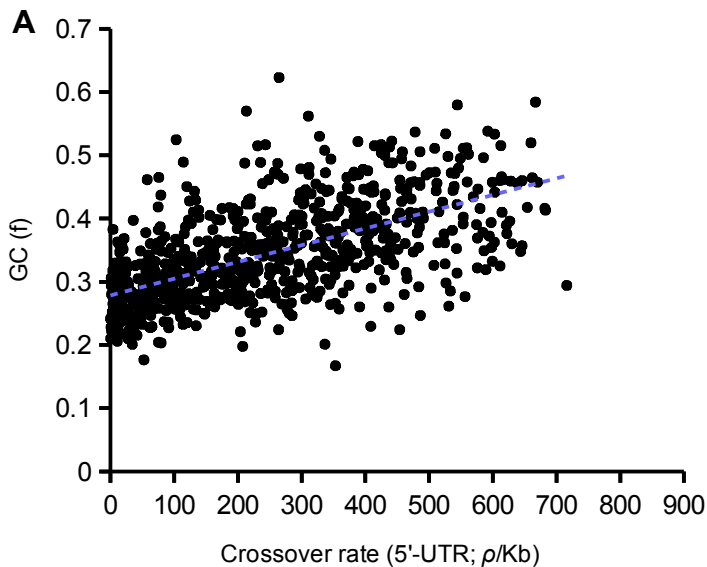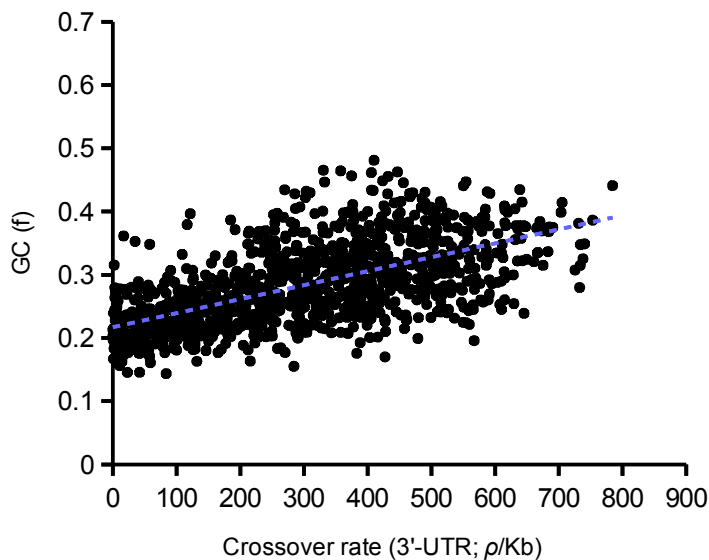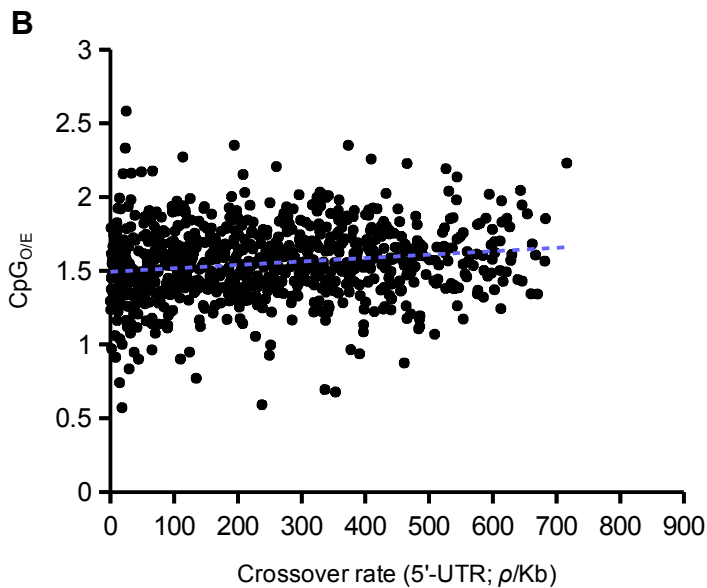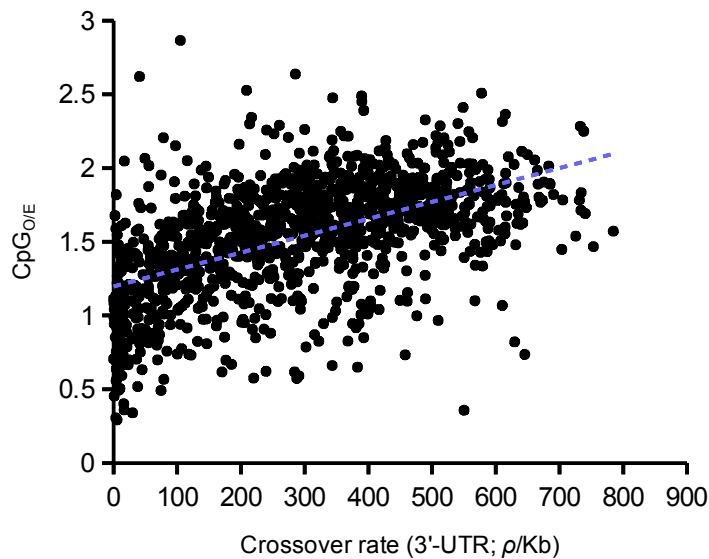

Supplement: S5 Fig — (A) Correlations between recombination rate and GC content in 5’-UTRs (R2 = 0.371, p<10-5) and 3’-UTRs (R2 = 0.388, p<10-5). (B) Correlations between recombination rate and CpGO/E in 5’-UTRs (R2 = 0.025, p<10-5) and 3’-UTRs (R2 = 0.289, p<10-5). 100 kb genomic windows are used in each comparison. (PDF) [file pgen.1005189.s005.pdf]

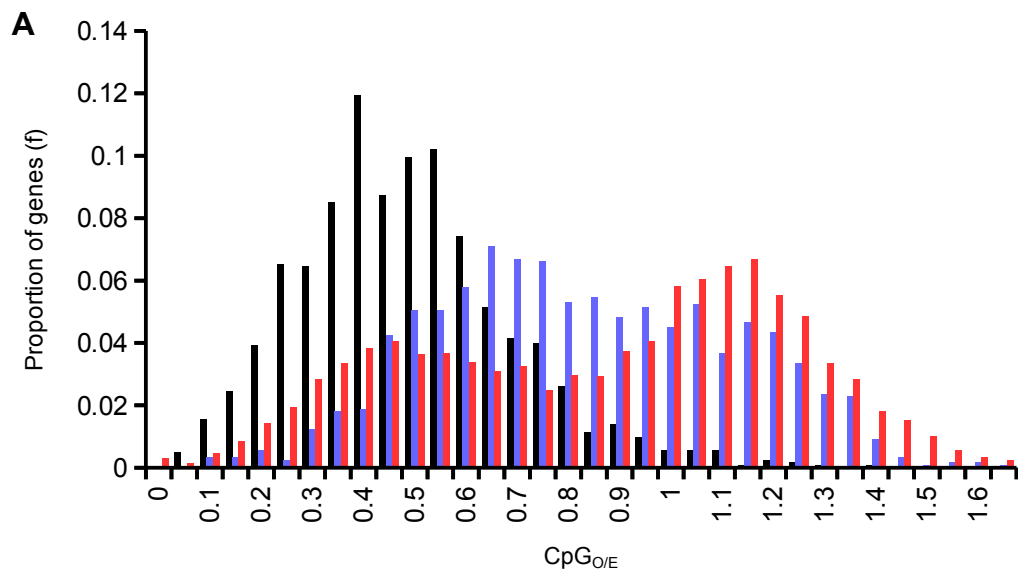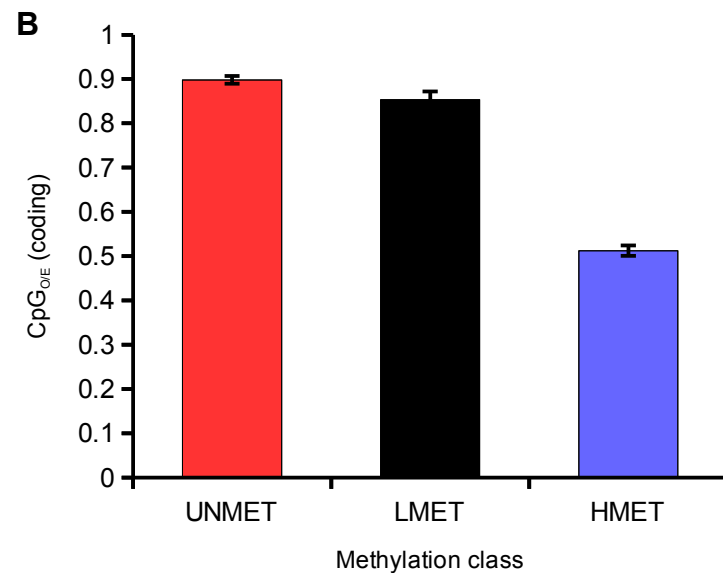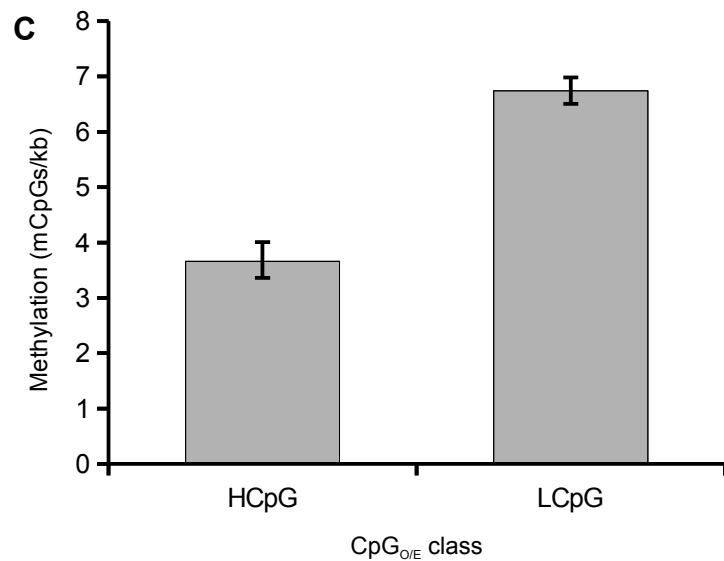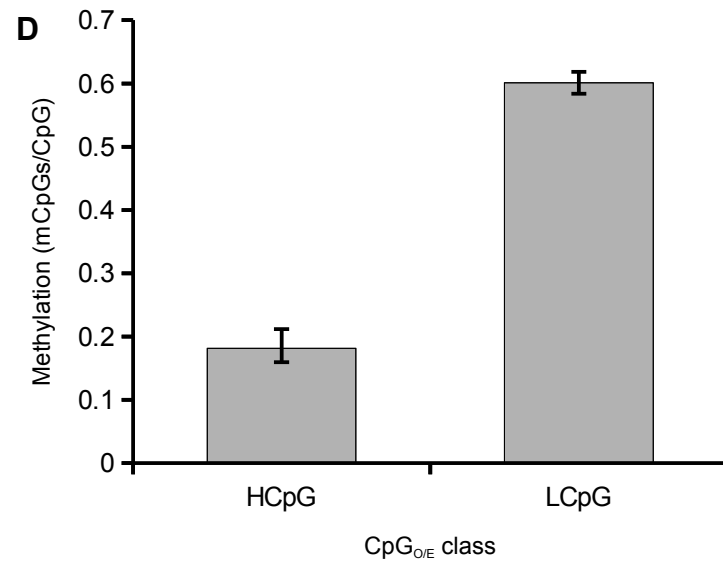

Supplement: S6 Fig — (A) Histogram of the genes grouped by CpGO/E values associated with each methylation class: unmethlylated (UNMET, red), high methylation (HMET, black) and low methylation (LMET, blue). The HMET distribution strongly deviates from the other classes, centering around low CpGO/E values. (B) Average CpGO/E values for genes within the three methylation classes (UNMET, LMET and HMET). (C) Average levels of methylation, measured in methylated CpGs / kb for HCpG and LCpG genes. (D) Average levels of methylation, measured in the proportion of CpGs that are methylated for HCpG and LCpG genes. 95% confidence intervals for B-D estimated from 200 bootstrap replicates. (PDF) [file pgen.1005189.s006.pdf]

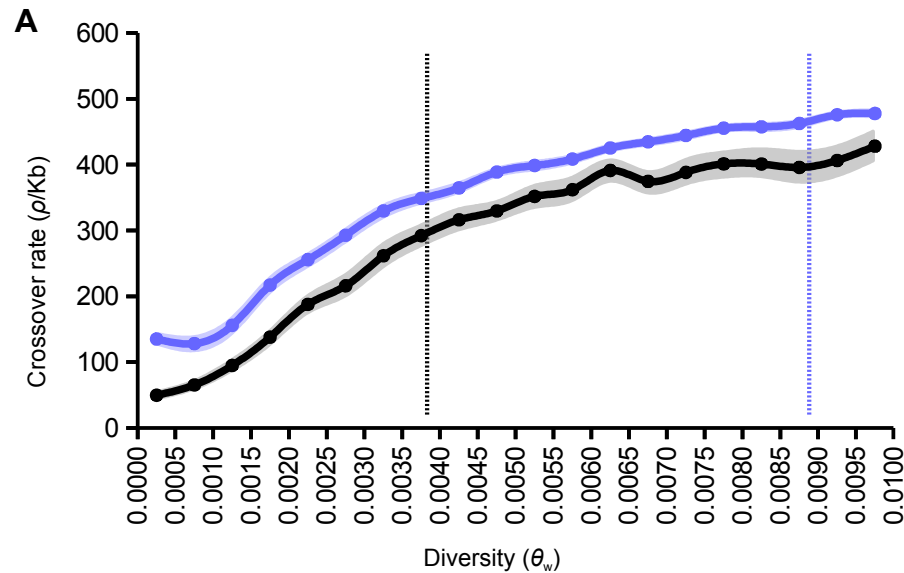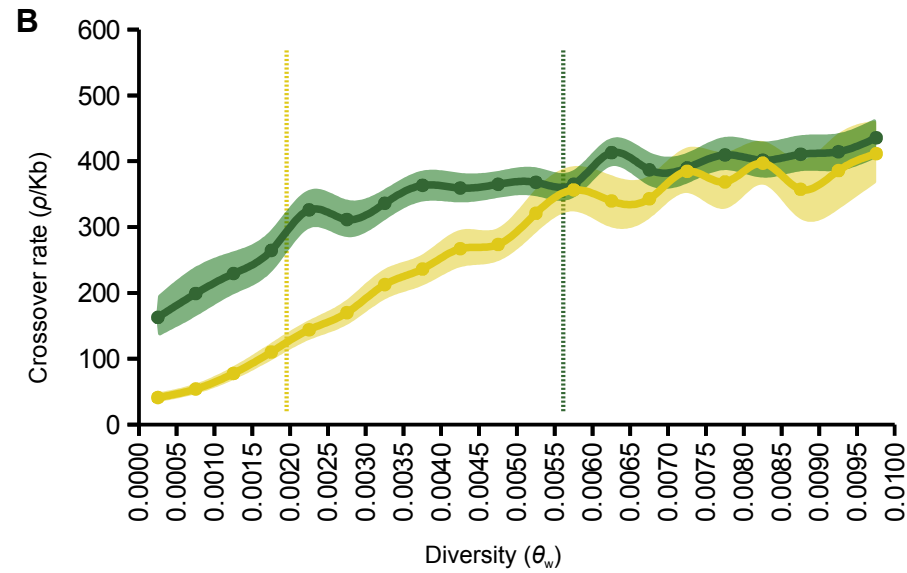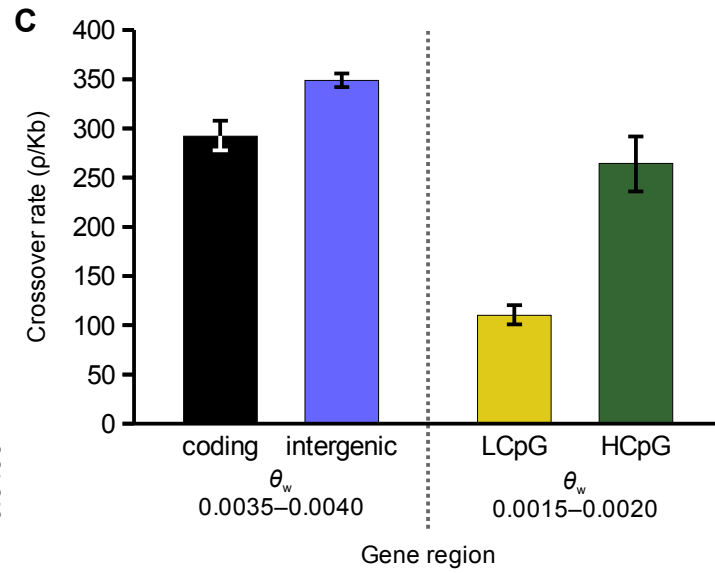

Supplement: S7 Fig — Crossover rates and diversity were measured in 1 kb windows across the genome. Windows spanning >500 bp of intergenic sequence were classified as intergenic regions. Windows spanning >500 bp of coding sequence were classified as coding regions and further subdivided according to the CpGO/E of the coding sequence (LCpG = CpGO/E<1.04; HCpG = CpGO/E>1.04). (A) Comparison of crossover rates between all coding and intergenic regions at given levels of genetic variation (dashed lines = mean genetic diversity of the region across all genes; shaded area = 95% confidence intervals generated from 200 bootstrap replicates of each interval). (B) Comparison of crossover rates between LCpG and HCpG coding regions at given levels of genetic variation (dashed lines and shaded areas as in A). (C) The subset of the comparisons include the mean levels of genetic diversity of all coding (θ w = 0.0038) and LCpG coding regions (θ w = 0.0020). (PDF) [file pgen.1005189.s007.pdf]

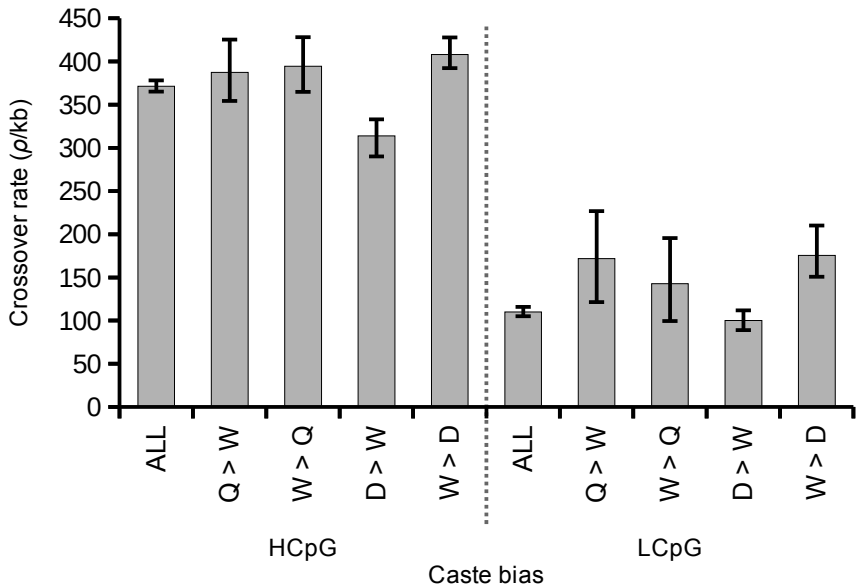

Supplement: S8 Fig — Average recombination rates of genes with caste biased expression (as in Fig 4) subdivided into classes of low (LCpG) or high (HCpG) CpG content. 95% confidence intervals generated from 200 bootstrap subsamples of all genes attributed to a particular gene class. (PDF) [file pgen.1005189.s008.pdf]

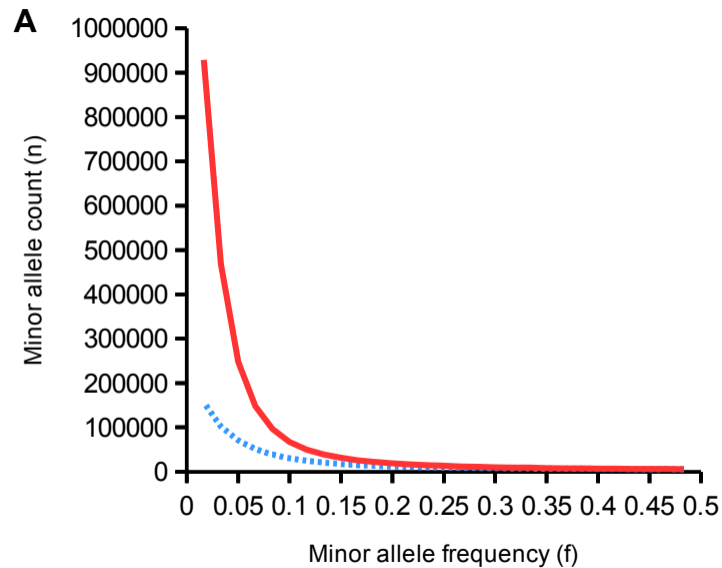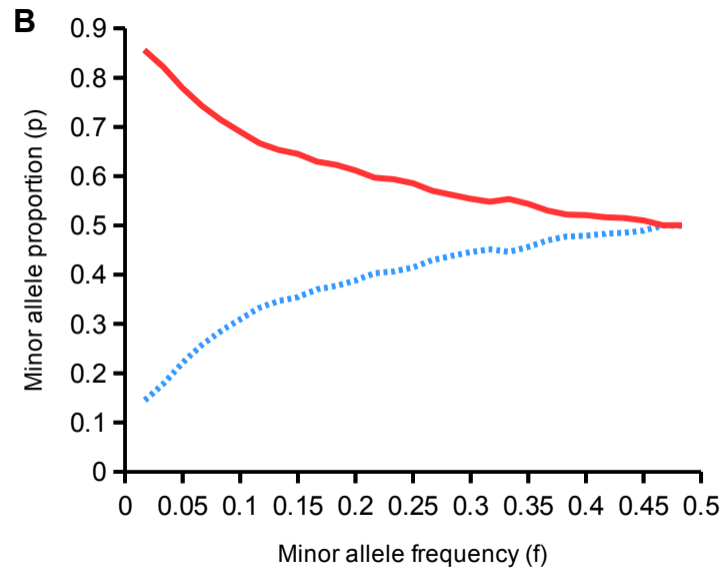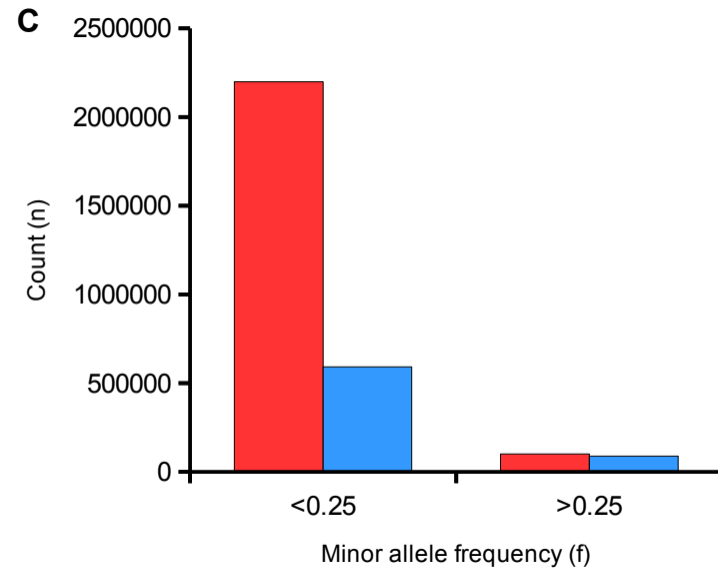

Supplement: S9 Fig — (A) The folded site frequency spectra computed from the minor allele frequencies of the 3M variants analysed in Fig 5 without polarizing the mutations using outgroup information. Minor allele variants are: W (A/T alleles; red line); S (G/C alleles; blue dashed line). (B) The relative site frequency spectra of the two minor allele variants (variants and colors as in A). (C) The number of W and S minor allele variants at low (<0.25) and intermediate (>0.25) frequencies, respectively (variants and colours as in A). There is a significant excess of S alleles segregating at intermediate frequencies (p<10-5; Fischer's exact test). (PDF) [file pgen.1005189.s009.pdf]

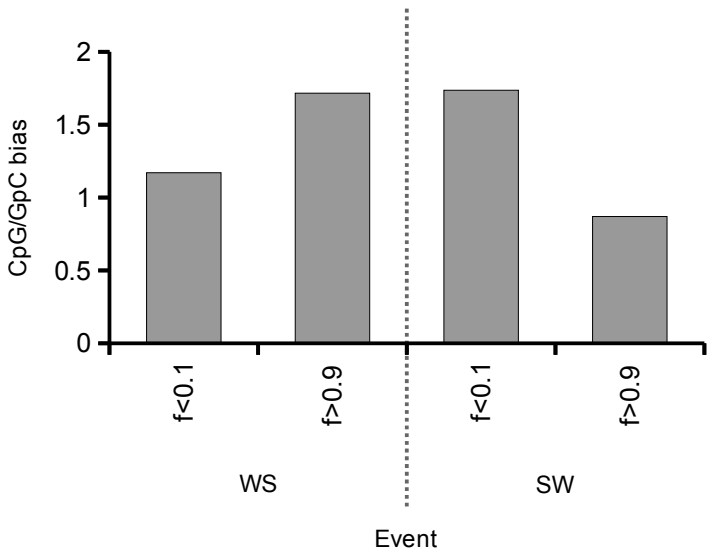

Supplement: S10 Fig — From left to right, the four bars show a) the proportion of WS variants that generate CpG sites compared with those that generate GpC sites at low derived allele frequencies (<0.1); b) the same ratio at high derived allele frequencies (>0.9); c) the proportion of SW variants at ancestral CpG sites compared with ancestral GpC sites at low derived allele frequencies (<0.1); d) the same ratio at high derived allele frequencies (>0.9). (PDF) [file pgen.1005189.s010.pdf]
