# Supplementary material for: Extreme Recombination Frequencies Shape Genome Variation and Evolution in the Honeybee, Apis mellifera
Source: PLoS Genet. 2015 Apr 22;11(4):e1005189. doi: 10.1371/journal.pgen.1005189 (PMC4406589; doi:10.1371/journal.pgen.1005189)
Supplement: S1 Text — (PDF) [file pgen.1005189.s013.pdf]

S1 Text: gBGC estimate and error rates

High gBGC may explain the pattern of polarization errors

When fitting the population genetics model of Glémin et al. (2014) we estimate very high polarization error probabilities for WS mutations (between 10 and 20%). This is the reverse to what is expected and found in humans where polarization errors are more frequent for SW mutations due to CpG hypermutability. Here, because the mutation bias towards AT seems to be very high (around 10) the same pattern should be expected. However, because gBGC is also very high, this could also generate homoplasy: a polymorphic WS mutation in the focal species can have also been fixed by gBGC in the outgroup species. It will thus be considered as a SW mutation in the focal species.

We can simply quantified this process by considering that the error rate is equal to the probability of substitution on the outgroup branch, which is the case under simple parsimony inference:

$$e_{SW} = \lambda u \frac{B}{e^{B}-1} t \qquad (1a)$$

and

$$e_{WS} = u \frac{B}{1-e^{-B}} t \qquad (1b)$$

where $B = 4N_e b$, u is the WS mutation rate, λ the mutational bias towards AT, and t the divergence time. We compare the predictions for human-like and bee-like data. We assumed $ut = 0.02$ for both species, $\lambda_{\mathrm{Human}} = 3$ and $\lambda_{\mathrm{Bee}} = 10$. If we consider an average B of 0.5 in human vs $B = 5$ in bee, we predict the observed patterns of a very high WS error rates in bee with $e_{WS} >> e_{SW}$ and $e_{WS} < e_{SW}$ in human (Figure A1.1)

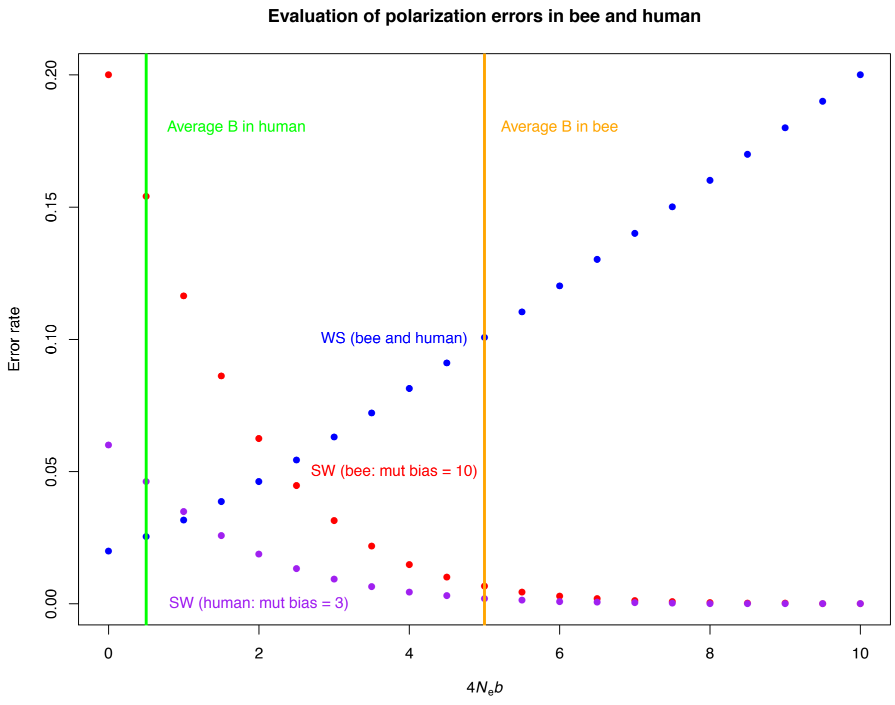

Figure A1.1

gBGC estimate is robust to high polarization error rates

Because some polarization error rates appear very high, we performed simulations to assess whether this could bias gBGC estimates. This was already done in Glémin et al. (2014) but using much lower error rates (compatible with human data). Here, we assumed that e_{Neutral} = 0.01, e_{SW} = 0.03 and e_{WS} = 0.15. We also assumed λ = 10 and that B linearly increased with GC content, from 1 to 20 (as estimated). For each GC-content value we simulated 100 datasets as in Glémin et al. (2014) and estimated B with and without error corrections (Figure A1.2). B is largely underestimated without error correction, as observed in the bee dataset, and accurately retrieved when error rates are taken into account in the model. The B estimates are thus robust to strong WS polarization errors.

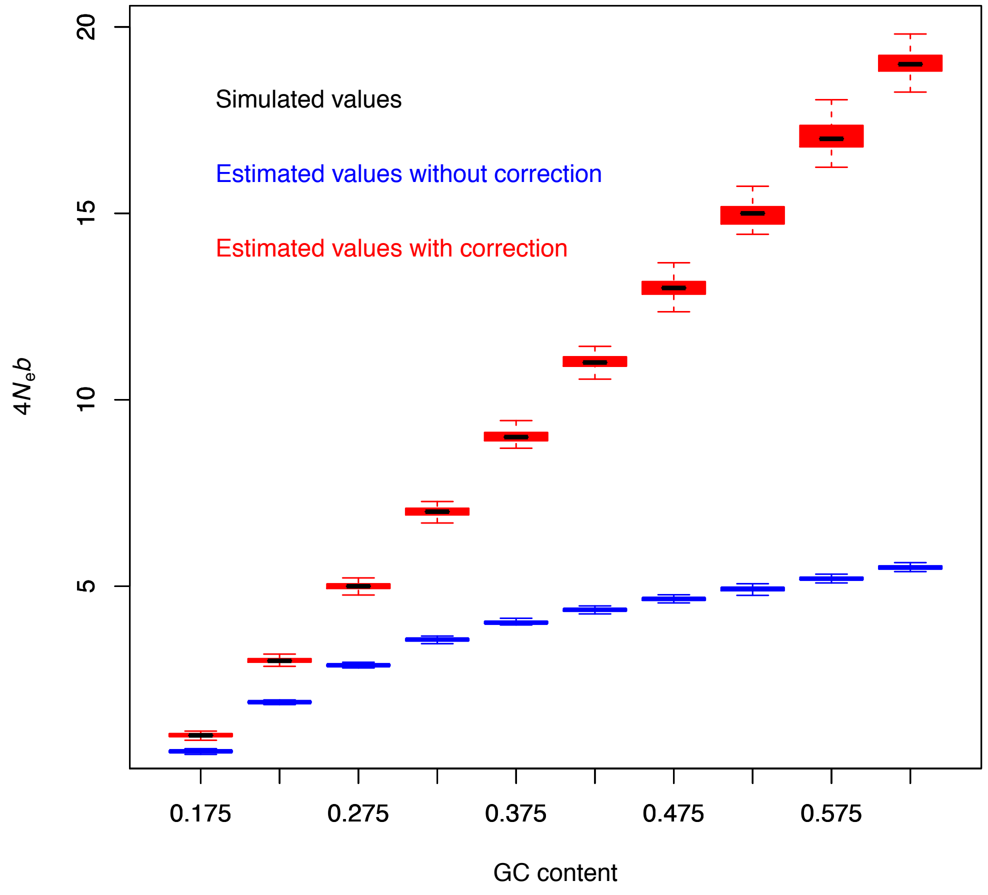

Figure A1.2
